# The butterfly effect in bisfluorenylidene-based dihydroacenes: aggregation induced emission and spin switching†
†Electronic supplementary information (ESI) available. CCDC 1947366–1947368. For ESI and crystallographic data in CIF or other electronic format see DOI: 10.1039/c9sc04096j


**DOI:** 10.1039/c9sc04096j

**Published:** 2019-10-07

**Authors:** Xiaodong Yin, Jonathan Z. Low, Kealan J. Fallon, Daniel W. Paley, Luis M. Campos

**Affiliations:** a Beijing Key Laboratory of Photoelectronic/Electrophotonic Conversion Materials , School of Chemistry and Chemical Engineering , Beijing Institute of Technology , Beijing 102488 , P. R. China; b Department of Chemistry , Columbia University , New York , New York 10027 , USA . Email: lcampos@columbia.edu

## Abstract

Linear acenes are a well-studied class of polycyclic aromatic hydrocarbons and their established physical properties have led to their widespread application across the field of organic electronics. However, their quinoidal forms – dihydroacenes – are much less explored and exhibit vastly different photophysical and electronic properties due to their non-planar, cross-conjugated nature. In this work, we present a series of difluorenylidene dihydroacenes which exhibit a butterfly-like structure with a quinoidal skeleton, resulting in comparatively higher optical gaps and lower redox activities than those of their planar analogs. We found that these compounds exhibit aggregation induced emission (AIE), activated through restriction of the “flapping” vibrational mode of the molecules in the solid state. Furthermore, anthracene-containing dihydroacenes exhibit thermally activated ground-state spin switching as evidenced by planarization of the acene core and diradical activity recorded by EPR. These two characteristics in this relatively unexplored class of materials provide new insights for the design of multifunctional materials.

## Abstract 2

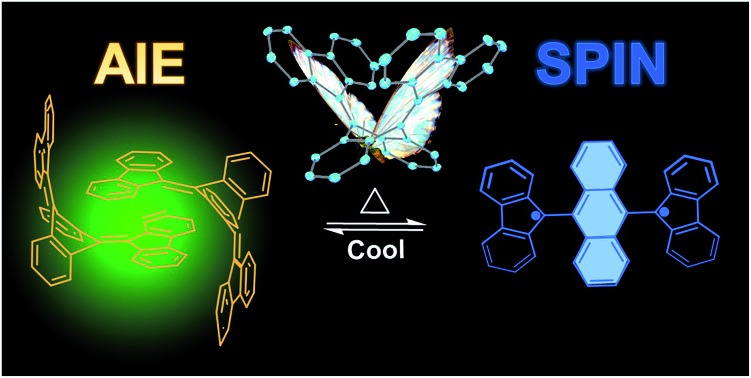
Difluorenylidene dihydroanthracene with a butterfly-like structure exhibits both aggregation induced emission (AIE) and thermally activated ground-state spin switching properties.

## Introduction

Polycyclic aromatic hydrocarbons (PAHs) are ubiquitous materials – they have been found in space^1^ and have made their way into a variety of optoelectronic devices^2^,^3^ and strong composite materials.^4^,^5^ Oligoacenes are a subclass of PAHs that have sparked interest in organic electronics^6^,^7^ due to the realization of their potential as active materials in organic field effect transistors (OFETs),^8^,^9^ organic light-emitting diodes (OLEDs),^10^,^11^ and third-generation photovoltaics.^12^–^16^ In most of these systems, fine-tuning the chemistry of the core structure and intermolecular interactions is essential for stability and the overall performance of the materials. These molecules are generally flat and exhibit strong pi-stacking interactions that can be beneficial or detrimental for particular types of applications. For example, aggregation can impair optical emission, but it can be beneficial for directional charge transport.^17^ In this vein, contorted PAHs can be tuned for practical applications. Additionally, introducing substituents with steric hindrance and conformational degrees of freedom can drastically alter their physical properties. For example, acenes with a butterfly structure were originally studied a couple of decades ago, and the butterfly structure can be changed to a flat structure by electrochemical oxidation.^18^–^21^

**Fig. 1:**
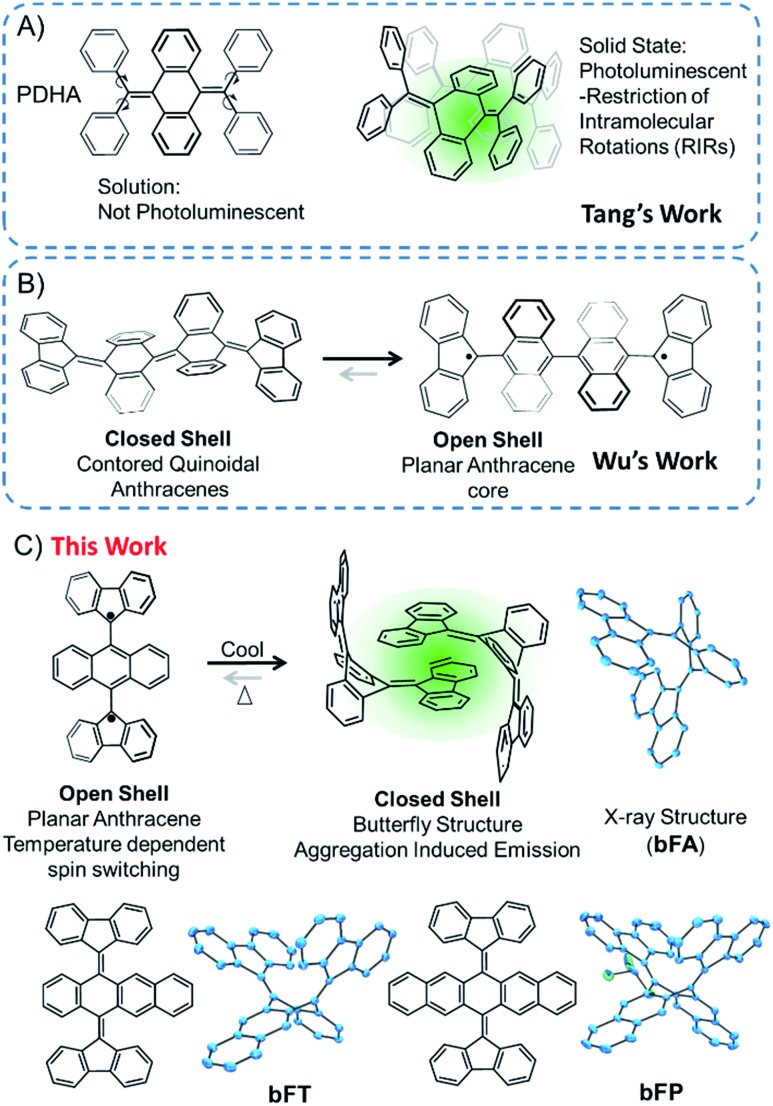
Schematic illustration of (A) Tang's work on aggregation induced emission properties of tetraphenyl dihydroanthracene (PDHA); (B) Wu's work on the diradical character of fluorene-capped Chichibabin's hydrocarbon; (C) AIE/spin dual mode switching properties of bisfluorenylidene dihydroanthracene (**bFA**) in this work.

Through precise molecular engineering of the core structure of oligoacenes, Tang and co-workers have shown that quinoidal derivatives of oligoacenes can exhibit aggregation induced restricted intramolecular vibration (RIV).^22^,^23^ AIE is counterintuitive since aggregation tends to quench photoluminescence of organic chromophores.^24^–^26^ For example, bis(diphenylmethylene)dihydroacenes are non-emissive in solution, but upon aggregation, they fluoresce in the solid state (Fig. 1A). This property does not translate to the fluoreno-quinoidal oligomers. In fact, fluoreno adducts are prone to stabilizing the radical character, as elegantly demonstrated by Wudl,^27^ Wu,^28^ Haley,^29^ and others.^30^,^31^ The fluoreno-derivative of Chichibabin's radical (Fig. 1B) is non-emissive; however, in its most stable structure, the diradical is readily characterized. This diradical character is not surprising, given that extending anthracene oligomers along the 9,10-positions disrupts pi-orbital overlap, thus forcing aromatization of the central ring and yielding the stable open-shell fluorenyl groups shown in Fig. 1B as the most favorable resonance structure. Such phenomena motivated our interest in investigating the structure–property relationship, extending the oligoacene core with bisfluoreno adducts. Interestingly, we found that the bisfluorenoanthracene in Fig. 1C exhibits both AIE in the solid state and the thermally accessible open-shell diradicaloid character.

The main focus of this study lies in understanding the fine interplay between the optical and magnetic properties by varying the aromaticity of the bisfluoreno-quinoidal core through the number of fused aromatic rings (Fig. 1C), **bFA** (anthracene core), **bFT** (tetracene) and **bFP** (pentacene). Surprisingly, we found that these compounds exhibit properties of both systems described above – AIE and switchable diradical character. The AIE fluorescence quantum yield was found to be up to 180 times higher for **bFP** in the solid-state than in solution. While AIE typically occurs due to RIR around the connecting bond of the flanking phenyl rings and the polyaromatic hydrocarbon core in aggregates or in the solid,^24^,^32^ the fluoreno-quinoidal molecules shown have no such degrees of freedom. These materials provide blueprints for a new class of luminogens where AIE arises solely from RIV of the strained butterfly-like structures. Moreover, the aromaticity of the quinoidal core is essential to drive the switching between low-spin (quinoid) and high-spin (planar oligoacene diradical) states. Notably, whilst spin switching is widely studied in transition metal complexes for applications in memory devices,^33^–^38^ pure hydrocarbon organic compounds with such properties are less common.^39^

## Results and discussion

Bisfluoreno-oligoacenes were synthesized from their corresponding acene-diones using the Corey–Fuchs reaction to generate *para*-bis(dibrominated) species,^40^ which were then reacted with 9,9-dimethyl-9-stannafluorene *via* Stille coupling to yield **bFA** (anthracene), **bFT** (tetracene) and **bFP** (pentacene) as colorless or light yellow crystals (see the ESI† for details). Single crystals of the three compounds were obtained by slow evaporation in a solution of chloroform/hexane (1 : 4 v/v), and the structures obtained by XRD are shown in Fig. 1C. The topology of all the molecules resembles butterfly shapes, with both fluorenes puckered on the same side.

The planes of the two fluorene moieties form a dihedral angle of 76.7° for **bFA**, 73.5° for **bFT** and 86.4° for **bFP**, respectively. The acene backbones of the molecules are bent at angles of 113.9°, 113.0° and 112.1° at the central ring for **bFA**, **bFT** and **bFP**, respectively. The bending angles of the acenes in this series are all more acute than those of the tetraphenyl derivatives, such as PDHA (Fig. 1), which bend at ∼132°. This is attributed to the increase in steric bulk of the fluorene moiety.^23^ As shown in Fig. S2,† the acene backbones also display a typical quinoidal structure – for example in **bFA**, C1–C15 and C8–C28 have double bond character (∼1.35 Å) while C1–C2, C1–C14, C8–C7, and C8–C9 have single bond character (∼1.49 Å). The molecules pack as dimers in the solid state where one fluorene group from each molecule engages in π–π stacking with the adjacent molecule at an average interplanar distance of ∼4.0 Å. The central fluorene groups in the dimer form a herringbone configuration, with a close CH–π interaction, ∼2.6 Å. The optimized gas phase structure of **bFA** determined by DFT (B3LYP/6-31G**) is similar to the crystal structure, except for a widening of the dihedral angle between the two fluorenes to 97.9°, which can be assigned to the intermolecular interactions in closely packed crystals. However, the bending angle of the central anthracene ring calculated to be 111.1° matches well with the crystallographic data.

During the synthesis and characterization of the molecules, we noticed remarkably bright fluorescent spots by thin-layer chromatography. Thus, we sought to probe the optical properties of **bFA**, **bFT** and **bFP** in solution (CH_2_Cl_2_). The results are summarized in Table 1 and Fig. S3.† All three compounds exhibit similar UV-Vis absorption spectra with an onset of ∼400 nm and maxima located at ∼350 nm. Unexpectedly, there is a slight blue shift in the absorption onset as the acene skeleton is extended from anthracene to pentacene, which was further confirmed by DFT calculations (B3LYP/6-31G**, see the ESI†). The electronic transition of the absorption maxima can be assigned to HOMO → LUMO (S_0_ → S_1_) for **bFA**, HOMO–1 → LUMO (S_0_ → S_2_) for **bFT** and HOMO–1 → LUMO and HOMO–1 → LUMO+1 (S_0_ → S_2_ and S_0_ → S_3_) transitions for **bFP**, respectively. Thus, we deduce that the blue shift in the spectra results from transitions to higher excited states in **bFP**.

**Table 1:** Summary of photophysical properties of difluorenylidene dihydroacenes

Entry	*λ*_abs_ (sol)^*a*^/(solid)^*b*^/nm	*λ*_em_ (sol)^*a*^	*λ*_em_ (solid)^*b*^	*Φ*_sol_ ^*a*^	*Φ*_agg_ ^*b*^	*τ*/ns (sol)	*τ*/ns (solid)
**bFA**	418/441	409, 431	512	0.8%	11.2%	0.30	8.84
**bFT**	413/440	513	511, 547	0.6%	24.9%	0.29	8.49
**bFP**	413/438	474	513	0.1%	18.5%	1.11	6.00

^*a*^In DCM solution; absolute quantum yields of these compounds were determined with an integrating sphere (Horiba Fluorolog-3).

^*b*^Thin film on a quartz slide; absolute quantum yields of these compounds were determined with an integrating sphere (Horiba Fluorolog-3).

**Fig. 2:**
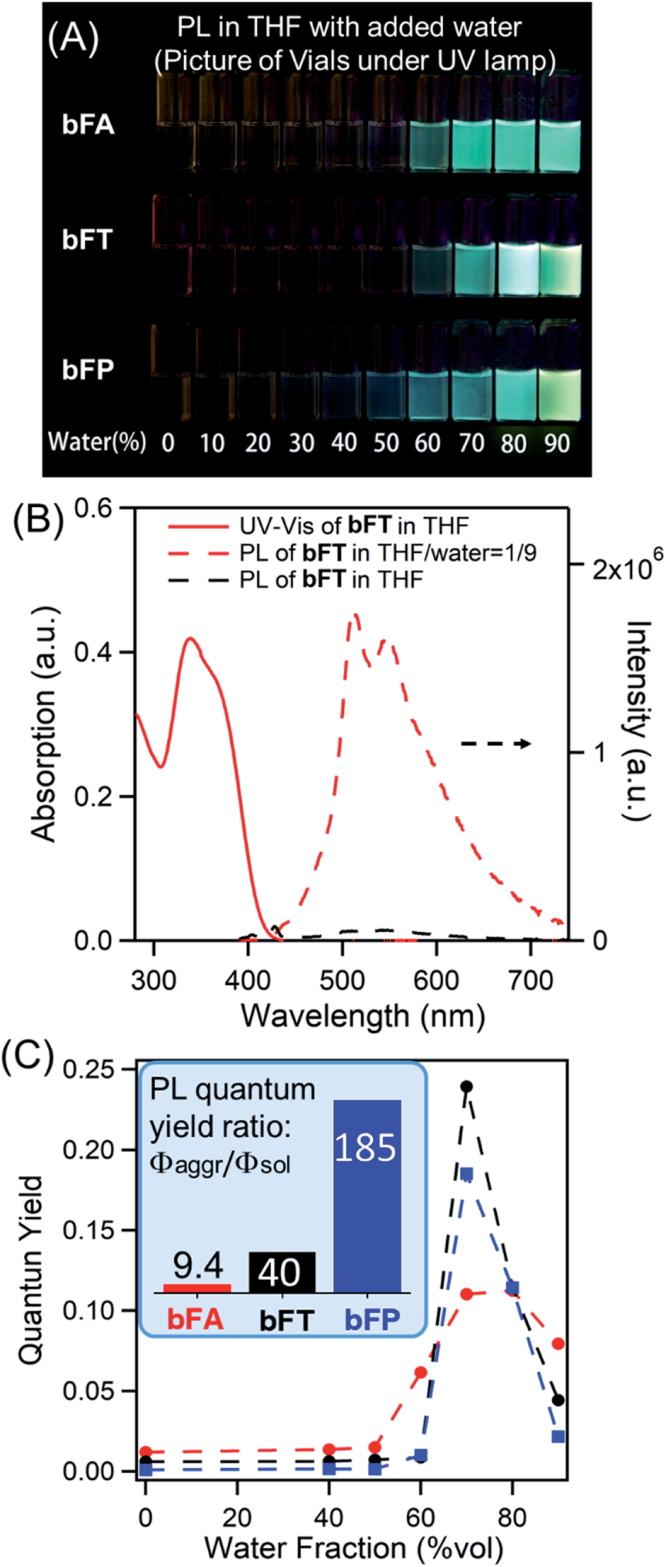
(A) Photographic image of quartz vials containing difluorenylidene acenequinoids displaying fluorescence in THF/H_2_O solutions with different water fractions irradiated using a 365 nm UV lamp; (B) UV-Vis and photoluminescence spectra of **bFT** in THF and THF/water = 1/9; (C) plots of quantum yield as a function of water fraction with excitation at 380 nm; the inset is the aggregation induced emission efficiency (*Φ*_aggr_/*Φ*_sol_); absolute quantum yields of these samples were determined with an integrating sphere (Horiba Fluorolog-3).

In solutions of dichloromethane and THF, all three compounds exhibit weak fluorescence, with quantum yields below 1% (Table 1). However, when an antisolvent (H_2_O) is introduced into THF solutions, fluorescence from the samples increases dramatically due to the formation of molecular aggregates. Fig. 2A shows a series of photos of **bFA**, **bFT**, and **bFP** in different THF/water mixtures, clearly demonstrating the increase in fluorescence as the percentage of water increases. Fig. 2B shows the UV-Vis and fluorescence spectra of **bFT** as an example, which indicate the dramatic increase of the fluorescence of **bFT** in THF/water = 1/9 in comparison with that in pure THF. The UV-Vis and fluorescence spectra (Fig. S4†) for all three compounds in solutions containing different percentages of water were recorded. In mixtures containing 10–50% water by volume, the emission intensity remains weak for all three compounds, indicating that they were well solvated. When the percentage of water reaches 60%, the emission intensity shows a sharp increase that is greater than two orders of magnitude. Furthermore, the fluorescence lifetime (*τ*) in the solid state is longer than that in solution for all molecules, and in **bFA** and **bTA** dramatically so (Table 1 and Fig. S5†).

Combining the UV-Vis and fluorescence spectra, the quantum yield of the three compounds can be plotted against the percentage of water (Fig. 2c). The extent of the enhancement arising from aggregation-induced emission (*α*_AIE_) can be defined as the quantum yield ratio of the aggregates to the solution (*Φ*_aggr_/*Φ*_sol_). As a guide, *α*_AIE_ ∼344 is seen in tetraphenyl ethylene (TPE), a typical AIE chromophore.^41^ In **bFA**, **bFT** and **bFP**, *α*_AIE_ is 9.4, 40 and 185, respectively (Fig. 2c). The observed AIE in all three compounds is remarkable given that there are no rotational degrees of freedom, which is generally the key mechanism by which AIE occurs in conventional systems.

**Fig. 3:**
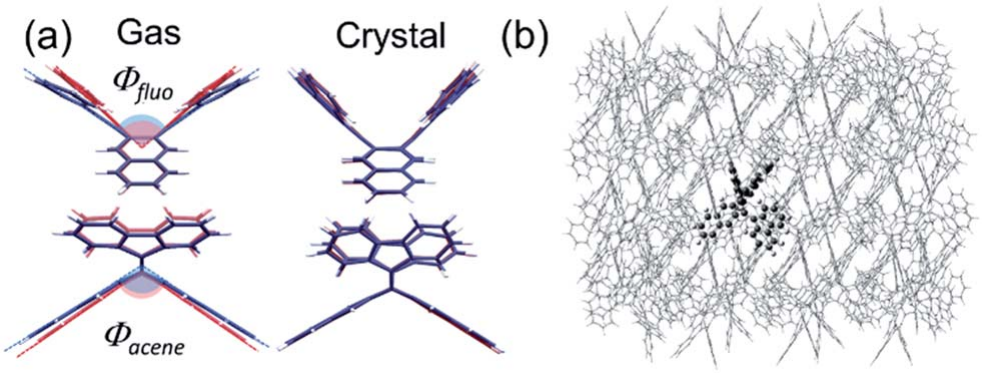
(a) Optimized structures of the ground state (S_0_, red) and excited state (S_1_, blue) conformations of **bFP** in the gas and crystal state with the B3LYP/6-31G** and ONIOM (B3LYP/6-31G**: UFF) methods, respectively; (b) the ONIOM model, with the high layer presented as ball-and-stick molecules and the low layer presented as wireframe molecules.

Here, the widely invoked restricted intramolecular rotation (RIR) model in AIE^22^ cannot explain our observations. Thus, we turn to density functional theory (DFT) calculations to investigate the origin of the observed AIE. We first calculated the ground and excited state structures of the compounds in the gas phase using the Becke3–Lee–Yang–Parr level of theory with a 6-31G** basis set (B3LYP/6-31G**). Fig. 3a shows that for **bFP**, the bending angles of both the acene core (*θ*_acene_) and the fluorenes (*θ*_fluo_) are more relaxed toward planarity in the excited state (S_1_, blue) than in the ground state (red) by 6° and 17°, respectively. We compare these results to calculations confined within the crystal structure as shown in Fig. 3b, obtained using a combined quantum mechanics/molecular mechanics (QM/MM) method, ONIOM (B3LYP/6-31G**: UFF), which is a reliable method of obtaining molecular structure information, embedded within a crystalline lattice (see details in the ESI†).^23^ The result shows that the ground state and excited state structures of **bFP** in the crystal are practically identical. Table S2† summarizes the computational results arising from structural changes for all three compounds under investigation. In all cases, a similar trend is observed where the ground state is more bent than the excited state in the gas phase, while both conformations are similar in a crystalline solid. The DFT results indicate that the AIE may result from restricted intramolecular vibrations (RIVs) only. This type of AIE has been observed before, but with only a slight enhancement in *α*_AIE,_*e.g.*, *ca.* 60 for **BDBA**^41^ and *ca.* 30 for **COTh-TMS**^42^ with all carbon atoms sp^2^ hybridized. Our results are comparable to changes in the quantum yield reported for AIE in chromophores that exhibit marked conformational changes, such as RIR.^43^ These results suggest that AIE may be common for other chromophores where RIR is absent, but RIV may result in unprecedented PL in the solid state.

**Fig. 4:**
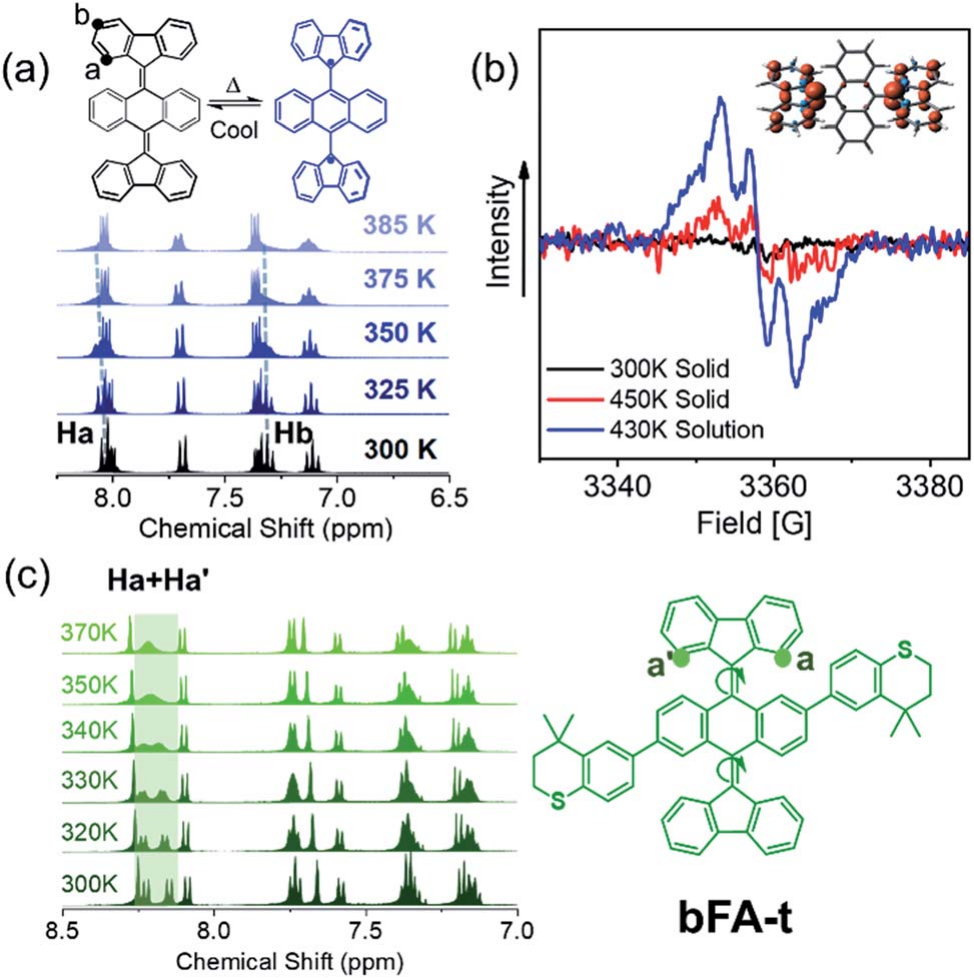
(a) VT-^1^H NMR of **bFA** in tetrachloroethane-d2; (b) EPR data of **bFA** in the solid state at 300 K (black) and 450 K (red) and in dichlorobenzene (4 × 10^–2^ M) at 430 K (blue); inset: spin density illustration of the T_1_ state of **bFA_twist_** (twisted configuration) at the UM06L/6-311G** level of theory; (c) VT-^1^H NMR of **bFA-t** and the molecular structure of **bFA-t**.

Considering that these chromophores have resonance structures where the quinoidal structures can flatten and rehybridize into the open-shell diradicals, we sought to investigate the temperature-dependent structure–property relationship between the closed-shell and open-shell ground state structures (Fig. 1C).^28^,^44^ Fig. 4a shows the ^1^H-NMR spectrum of **bFA** as a function of temperature varying from 300 K to 385 K. We observe that the peaks of the H_a_ doublet (8.1 ppm) and H_b_ triplet (7.2 ppm, which overlaps with the solvent peak and a quartet) broaden considerably when the solution is heated beyond 373 K. Furthermore, electron paramagnetic resonance (EPR) spectroscopy performed at 430 K in dichlorobenzene shows a signal that matches well with previous reports of radicals localized on fluorene moieties (Fig. 4b).^28^,^45^ EPR of a solid sample of **bFA** was also obtained, but show much weaker signals even at a high temperature (450 K). Taken together, the NMR and EPR results indicate that **bFA** adopts an open shell configuration at high temperatures, forming a triplet diradical.

To gain insight into the structural changes of the **bFA** backbone upon conversion to the open diradical, we synthesized an asymmetric analog, **bFA-t**, so that the signal from H_a_ and H_a′_ protons would be split into two distinct doublets, as shown in Fig. 4c. These doublets appear at 8.0–8.2 ppm. Fig. 4c also shows that as the temperature is increased, the signals from H_a_ and H_a′_ coalesce, indicating that the fluorene moiety is able to rotate freely. The free rotation of the fluorene group supports the formation of an open-shell diradical, since the double bond between fluorene and acene gains significant single bond character during this transformation. From the NMR data shown in Fig. 4c, we calculate that the energy barrier of the rotation (Δ*G*^≠^) in **bFA-t** is 17.3 kcal mol^–1^ (Fig. S11†). In contrast to **bFA**, neither **bFT** nor **bFP** show line broadening of the proton signals as the temperature is increased; rather, the signals are sharpened, and no signal of EPR was observed (see Fig. S12†), indicating that these molecules do not have a thermally accessible open-shell ground-state configuration. Besides, the barrier to rotation (Δ*G*^≠^) for **bFT** is estimated to be *ca.* 21.7 kcal mol^–1^ by the NMR data (Fig. S13†), which is much higher than that of **bFA-t**.

It is noteworthy to report our observation of a diprotonated derivative of **bFA-t** (**bFAH-t**) with strong blue fluorescence as the main product of the microwave-assisted Stille coupling reaction at ∼180 °C (see Fig. S14†). This is strong evidence of H-abstraction of a diradical intermediate of **bFA-t** at high temperature.^46^,^47^ The UV-Vis absorbance of **bFAH-t** indicates that the core of this molecule has an aromatic anthracene structure with a finely split peak at *ca.* 400 nm. Moreover, this diprotonated compound can be converted back to the quinoid structure by oxidation with DDQ, as shown in Fig. S15.†

**Fig. 5:**
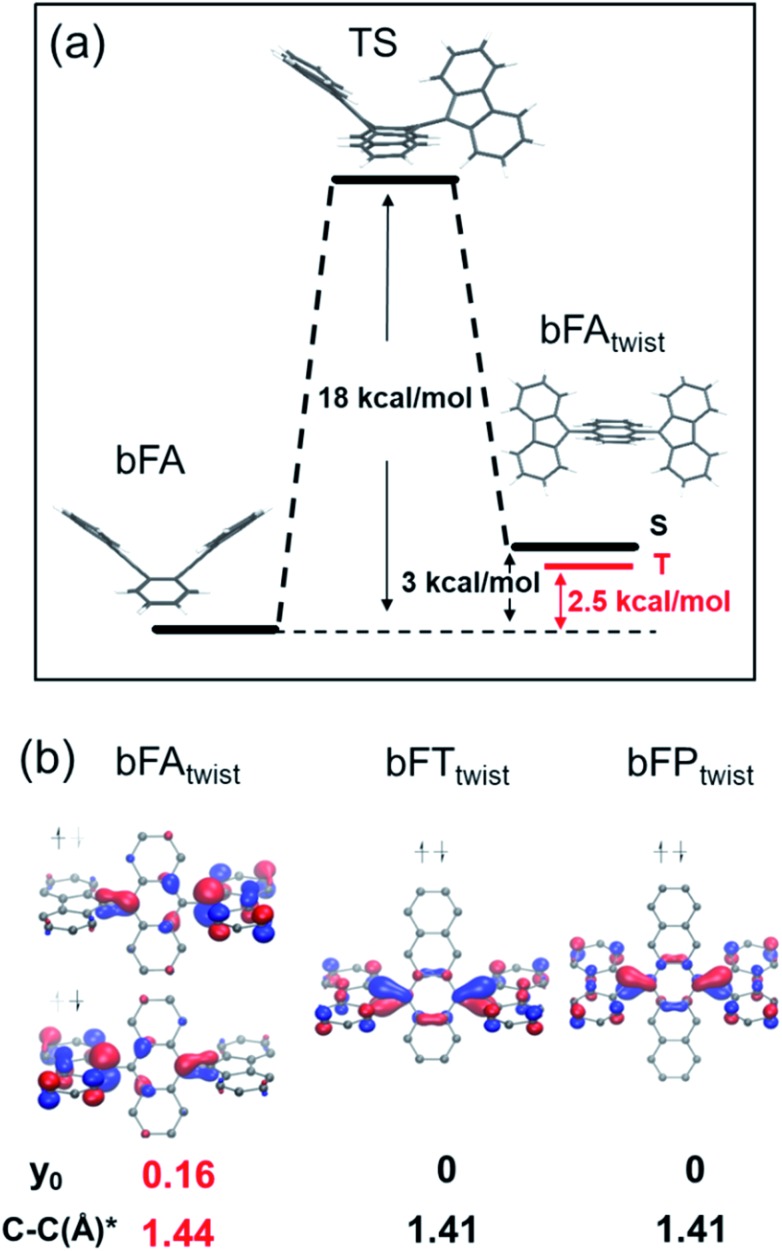
(a) Energy diagram of **bFA** with rotation of the fluorene moiety; (b) HOMOs of the fluorene twisted structure of **bFA** (**bFA_twist_**), **bFT** (**bFT_twist_**), and **bFP** (**bFP_twist_**), with the *y*_0_ value and bond length (*) of the C–C bond between fluorene and acenes.

In order to understand the spin switching process, we computed the stationary points and transition state for **bFA**, as it changed into the open-shell structure from the closed-shell ground state (DFT, UB3LYP/6-31G**, Fig. 5A). As the dihedral angle joining fluorene to anthracene was planarized, an energy saddle point was observed in the conformation where one fluorene is twisted relative to anthracene whilst the other one is not. The theoretical energy barrier of 18 kcal mol^–1^ compares well with the experimental result of deuterium-NMR of **bFA-t** with the same aromatic core (see Fig. S11†). When both fluorenes are twisted, a relatively stable state is formed with a comparable energy level (+3 kcal mol^–1^) to that of the original state. Single point energy calculations of both the single twisted structure and double twisted structure were conducted at the UM06L/6-311G** level of theory.

The biradical character of organic materials can be assessed by the biradical character index (*y*_0_) and indicates the contribution of the open-shell resonance form to the overall ground state structure, ranging from a pure biradical (*y*_0_ = 1) to a purely closed shell (*y*_0_ = 0).^48^–^51^ For **bFA**, *y*_0_ values of 0 and 0.16 were obtained for the single twisted structure and double twisted structure, respectively, indicating the open-shell character of the double twisted structure (**bFA_twist_**) as shown in Fig. 5b. In addition, the theoretical C–C double bond length in **bFA_twist_** between the fluorene and anthracene moieties shows a remarkable extension (1.44 Å) compared to that in the butterfly configuration (1.36 Å), indicating the open shell character from a structural point of view. Crucially, the triplet state of **bFA_twist_** is calculated to be lower in energy than the diradical singlet state (as shown in Fig. 5), indicating that intersystem crossing is thermodynamically favorable in this compound. The spin density of the T_1_ state of **bFA_twist_** is mainly distributed on the fluorene moiety, especially on carbons a and b, which match very well with the signal broadening observed by VT-^1^H NMR (see Fig. 4b). Theoretical analysis was also conducted for the twisted structures of **bFT** (**bFT_twist_**) and **bFP** (**bFP_twist_**), and both of them exhibit a closed shell ground state electronic structure (*y*_0_ = 0, see Fig. 5b). These calculations match well with our experimental observations. Furthermore, the carbon–carbon bond length between fluorene and acenes of **bFT_twist_** and **bFP_twist_** is *ca.* 1.41 Å which is more comparable to a typical double bond, indicating closed-shell quinoidal structures.

From this analysis we conclude that, at elevated temperatures, aromatization is a strong enough driving force to access the open shell ground state in the anthraquinoid **bFA**, whereas the longer acenequinoid structures in **bFT** and **bFP** do not possess sufficient aromatic character in their aromatic conformation to exhibit this trait. This finding is supported by Clar's theory of aromatic sextets, where higher order acene molecules exhibit greater stability as two small acenes *versus* one large acene system (*e.g.* 2 × naphthalene *vs.* 1 × pentacene for **bFP**).^52^,^53^ Besides, since the open-shell character of **bFA** is strongly related to the rotation of fluorene moieties, the weak EPR signal in the solid state even at a high temperature can be assigned to the constriction of intramolecular motion.

## Conclusions

In summary, we synthesized a series of difluorenylidene dihydroacene compounds using a facile and efficient method. These compounds exhibit wider optical gaps than expected due to their bent, butterfly-like quinoid structures. All compounds exhibit aggregation induced emission, which is rarely seen in such a kind of structure without any freely rotatable functionalities. A flapping intramolecular vibration was identified as the mechanism based on theoretical analysis (QM/MM calculations). Furthermore, a transformation between the bent quinoid butterfly configuration and the aromatic planar configuration is thermally accessible in the anthracene-containing material. As a result, the electronic structure is also switchable between the closed shell at a low temperature and open shell at a high temperature, which is confirmed by VT-^1^H NMR and EPR. This phenomenon can be assigned to rotation of the fluorene moieties at a high temperature, leading to an intermediate structure with a flattened anthracene skeleton and moderate diradical character. Conversely, this transition is not thermally accessible in longer acenequinoids, due to the lower driving force as a function of the lower aromaticity of the aromatic conformation of these acenes. We envisage that the structure–property relationship reported herein will create new pathways for designing novel multi-functional materials.

## Conflicts of interest

There are no conflicts to declare.

## Supplementary Material

Supplementary information

Crystal structure data
