# Supplementary material for: The butterfly effect in bisfluorenylidene-based dihydroacenes: aggregation induced emission and spin switching
Source: Chem Sci. 2019 Oct 7;10(46):10733–9. doi: 10.1039/c9sc04096j (PMC7020927; doi:10.1039/c9sc04096j)
Supplement: Supplementary file 1 [file SC-010-C9SC04096J-s001.pdf]

Electronic Supplementary Material (ESI) for Chemical Science.
This journal is © The Royal Society of Chemistry 2019

Electronic Supplementary Information

The Butterfly Effect in Bisfluorenylidene-based Dihydroacenes: Aggregation Induced Emission and Spin Switching

Xiaodong Yin[a, b], Jonathan Z. Low[b], Kealan J. Fallon[b], Daniel W. Paley[b], and Luis M. Campos*[b]

[a]Beijing Key Laboratory of Photoelectronic/Electrophotonic Conversion Materials, School of Chemistry and Chemical Engineering, Beijing Institute of Technology, Beijing 102488, P.R.China

[b]Department of Chemistry, Columbia University, New York, New York 10027, United States.

Index

S1. Materials and methods 1

S2. Experimental Section 2

S3. Crystal Structure 7

S4. Supporting Data 10

S5. DFT Calculation 17

S6. Characterizaiton Data 22

S7. References 33

S1. Materials and methods

General Methods: General Methods: All commercially obtained reagents/solvents were used as received; chemicals were purchased from Alfa Aesar®, Sigma-Aldrich®, Acros organics®, TCI America®, Mallinckrodt®, and Oakwood® Products, and were used as received without further purification. Unless stated otherwise, reactions were conducted in oven-dried glassware under argon atmosphere. Anhydrous solvents were obtained from a Schlenk manifold with purification

columns packed with activated alumina and supported copper catalyst (Glass Contour, Irvine, CA). All reactions were carried out under argon unless otherwise noted.

^{1} H-NMR and ^{13}C-NMR spectra were recorded on Bruker 400 MHz and on 500 MHz (125 MHz for ^{13}C) spectrometers. Data from the ^{1}H-NMR and ^{13}C spectroscopy are reported as chemical shift (δ ppm) with the corresponding integration values. Coupling constants (J) are reported in hertz (Hz). Standard abbreviations indicating multiplicity were used as follows: s (singlet), b (broad), d (doublet), t (triplet), q (quartet), m (multiplet) and virt (virtual).

The VT-^{1}HNMR were recorded on Bruker 300 MHz and 500MHz spectrometer. DNMR data was simulated with TopSpin 3.5 software to obtain exchange rate "k", then $\Delta G^{\neq}$ can be generated from the equation:

$$\Delta G^{\neq} = 1.987\ T\,(23.760+\ln(T/k_r))$$

The mass spectral data for the compounds were obtained from XEVO G2-XS Waters® equipped with a QTOF detector with multiple inlet and ionization capabilities including electrospray ionization (ESI), atmospheric pressure chemical ionization (APCI), and atmospheric solids analysis probe (ASAP). The base peaks were usually obtained as $[M]^+$ or $[M+H]^+$ ions.

Cyclic voltammograms (CVs) were recorded on a CH166 electrochemical workstation using Ag/AgCl electrode as the reference electrode at room temperature. Experiments were performed in dry CH_2Cl_2 with NBu_4PF_6 as the supporting electrolyte at a scan rate of 0.1 V/s.

DFT calculations were conducted with Gaussian 09 (Rev. D 01) package.1 The input files were generated from single crystal structures when available or otherwise generated in Chem3D and then pre-optimized in Spartan '12 V 1.2.0. Geometries were then optimized in Gaussian09 using the hybrid density functional B3LYP with a 6-31G** basis set. Frequency calculations were performed to confirm the presence of local minima (positive frequencies). The orbital energy levels were calculated by single point calculations using the B3PW91 functional with a 6-311+G* basis set (the larger basis set gave more reasonable results for the dimeric species in comparison to the monomers). Vertical excitations were then calculated using TD-DFT methods (B3LYP/6-31G**). Orbital representations were plotted with Gaussview 5.08 (scaling radii of 75%, isovalue of 0.02).

S2. Experimental section

Scheme S1. Synthetic route of difluorenylidene dihydroacenes compounds

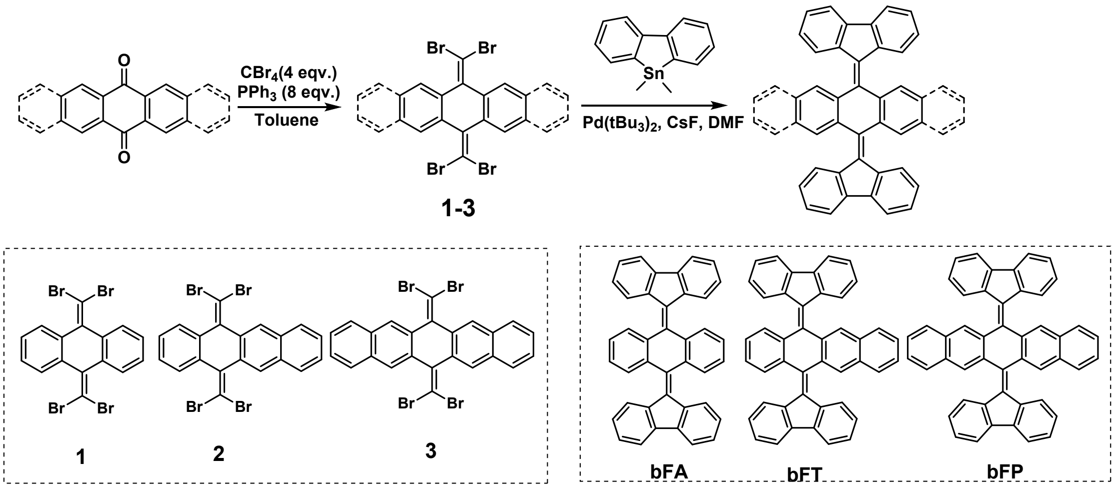

9, 10-bis(dibromomethylene)-9,10-dihydroanthracene (1)[2], **5, 12-bis(dibromomethylene)-5, 12-dihydrotetracene (2)**[3] and **6, 13-bis(dibromomethylene)-6, 13-dihydropentacene (3)**[4] were synthesized follow literatures.

9, 10-di(9H-fluoren-9-ylidene)-9, 10-dihydroanthracene (bFA) Compound **1** (0.5 g, 0.96 mmol) and 5,5-dimethyl-5H-dibenzo[b,d]stannole (0.63 g, 2.1 mmol) was added into a pre-dried schlenk flask (50 mL). This flask was degassed and filled by argon via pump-refill cycles 3 times, then 25 mL dry DMF was added. The mixture was stirred under argon for 15 mins, then cesium fluoride (CsF) (0.32g, 2.1 mmol) and $Pd(tBu_3P)_2$ (53.6 mg, 0.105 mmol) were added. The flask was heated up to 100 °C for 24 h, then cooled to room temperature. The mixture was diluted with 100 mL DCM, and then washed with water (3×100 mL). The organic phase was collected, and dried over Sodium Sulfate anhydrous. The solvent was removed by rotor vapor, and the residue solid was further purified by silica gel column (Hexanes : DCM = 5:1) to obtain pure product as light yellow solid 0.36g (75%).

^{1}H NMR (500 MHz, $CDCl_3$) δ 8.04 (d, J = 7.9 Hz, 2H), 7.97 (dd, J = 5.6, 3.3 Hz, 2H), 7.67 (d, J = 7.5 Hz, 2H), 7.30 (dd, J = 5.5, 3.1 Hz, 2H), 7.27 (t, J=6.5 Hz, 2 H) 7.07 (t, J = 7.6 Hz, 2H).

13C NMR (126 MHz, $CDCl_3$) δ 141.05, 140.27, 138.51, 138.00, 130.82, 128.94, 128.51, 126.51, 126.36, 125.78, 119.62, 77.45, 77.20, 76.95.

Hi-res Mass Spectrum [M+H$^+$] $C_{40}H_{25}$ Cald: 505.1956 Found: 505.1968

5, 12-di(9H-fluoren-9-ylidene)-5, 12-dihydrotetracene (bFT) Compound **2** (0.5 g, 0.88 mmol), and 5,5-dimethyl-5H-dibenzo[b,d]stannole (0.58 g, 1.94 mmol), cesium fluoride (CsF) (0.29, 1.94

mmol) and $Pd(tBu_3P)_2$ (50 mg, 0.1 mmol). After silica gel column purification (Hexanes : DCM = 5:1) to obtain pure product as light yellow solid 0.37g (76%).

^{1}H NMR (400 MHz, CDCl3) δ 8.42 (s, 1H), 8.13 (d, J = 8.0 Hz, 1H), 8.05 (d, J = 8.0 Hz, 1H), 7.98 (dd, J = 5.7, 3.3 Hz, 1H), 7.84 (dd, J = 6.1, 3.3 Hz, 1H), 7.69 (d, J = 7.4 Hz, 2H), 7.55 (dd, J = 6.2, 3.2 Hz, 1H), 7.31 (dd, J = 5.7, 3.3 Hz, 1H), 7.12 – 7.05 (m, 1H), 7.04 – 6.97 (m, 1H).

^{13}C NMR (126 MHz, CDCl3) δ 141.16, 141.00, 140.29, 138.57, 138.47, 138.25, 137.33, 131.79, 130.95, 128.93, 128.54, 128.34, 127.61, 127.07, 126.92, 126.55, 126.43, 125.63, 125.50, 119.69, 119.65.

Hi-res Mass Spectrum [M+H$^+$] $C_{44}H_{27}$ Cald: 555.2113 Found: 555.2126

6, 13-di(9H-fluoren-9-ylidene)-6, 13-dihydropentacene (bFP)

Compound **3** (0.6 g, 0.97 mmol), and 5,5-dimethyl-5H-dibenzo[b,d]stannole (0.64 g, 2.13 mmol), cesium fluoride (CsF) (0.32, 2.13 mmol) and $Pd(tBu_3P)_2$ (54 mg, 0.11 mmol). After silica gel column purification (Hexanes : DCM = 5:1) to obtain pure product as light yellow solid 0.43g (73%).

^{1}H NMR (500 MHz, CDCl3) δ 8.43 (s, 4H), 8.13 (d, J = 8.0 Hz, 4H), 7.84 (dd, J = 6.2, 3.3 Hz, 4H), 7.71 (d, J = 7.4 Hz, 4H), 7.54 (dd, J = 6.3, 3.2 Hz, 4H), 7.28 (t, J = 6.5 Hz, 4H), 7.02 (t,J = 7.0 Hz, 4H).

^{13}C NMR (126 MHz, CDCl3) δ 141.12, 138.54, 138.49, 137.39, 132.04, 131.16, 128.60, 128.35, 127.61, 127.10, 126.61, 125.38, 119.72.

Hi-res Mass Spectrum [M+H$^+$] $C_{48}H_{28}$ Cald: 605.2269 Found: 605.2276

Scheme S2. Synthetic route of difluorenylidene dihydroacenes compounds

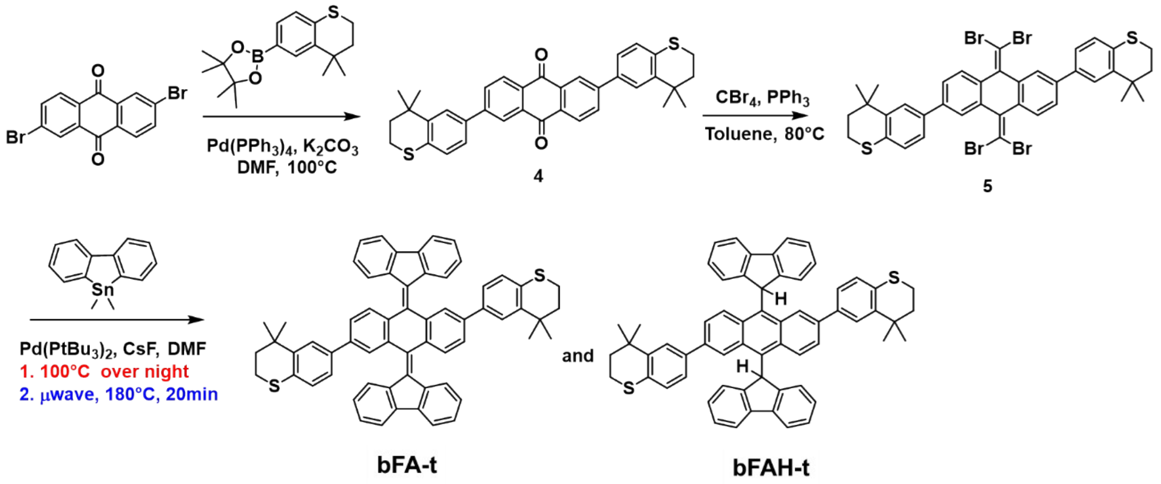

2,6-bis(4,4-dimethylthiochroman-6-yl)anthracene-9,10-dione (4)

2,6-dibromoanthracene-9,10-dione (1.2 g, 3.3 mmol) and 2-(4,4-dimethylthiochroman-6-yl)-4,4,5,5-tetramethyl-1,3,2-dioxaborolane (2.2 g 7.26 mmol) was charged into a 100 mL schlenk flask with dried DMF 50 mL, and the mixture was bubbled with nitrogen. After 20 min, Potassium Carbonate (K_2CO_3) 2.7 g suspended in 2 mL water was added into the flask, and bubbled with nitrogen for another 10 min, followed by addition of 230 mg of $Pd(PPh_3)_4$ (0.2 mmol). Then, the mixture was heated to 100 °C for 10 hrs. After the mixture was cooled to room temperature, 100 mL dichloromethane was added to dilute the mixture, and the mixture was washed with 200 mL of water and 200 mL of brine, respectively. The collected organic phase was dried over anhydrous sodium sulfate, the solvent was removed by rotorvapor, and the crude product was purified with silica gel column (Hexanes : DCM = 5:1) to obtained pure product was yellow solid 1.4 g (2.47 mmol, 75 %).

^{1}H NMR (500 MHz, $CDCl_3$) δ 8.51 (s, 2H), 8.38 (d, J = 8.1 Hz, 2H), 7.98 (d, J = 8.1Hz, 2H), 7.72 (s, 2H), 7.42 (d, J = 8.2Hz, 2H), 7.23 (d, J = 8.2 Hz, 2H), 3.13 – 3.06 (m, 4H), 2.06 – 1.98 (m, 4H), 1.43 (s, 12H).

^{13}C NMR (126 MHz, $CDCl_3$) δ 183.20, 147.06, 142.90, 134.86, 134.25, 133.87, 132.06, 131.95, 128.26, 127.49, 125.37, 125.10, 124.99, 37.58, 33.39, 30.32, 23.33.

Hi-res Mass Spectrum [M+H$^+$] $C_{36}H_{33}O_2S_2$ Cald: 561.1922 Found: 561.1923.

6,6'-(9,10-bis(dibromomethylene)-9,10-dihydroanthracene-2,6-diyl)bis(4,4-dimethylthiochromane) (5)

Compound **4** (1 g, 1.78 mmol) and 2.4 g of tetrabromocarbon (7.12 mmol), 3.7 g of triphenylphosphine (14.2 mmol) was charged into a 100 mL shlenk flask, followed with addition of 50 mL dried toluene. The mixture was bubbled with nitrogen for 20 min and heated to 90°C for 10 hrs. After the mixture was cooled to room temperature, it was filtered, and washed with toluene (50 mL). The collected filtrate was dried over anhydrous sodium sulfate, the solvent was removed with rotorvapor, and the crude product was purified with silica gel column (hexanes : DCM = 10 :1) to obtain pure product as white solid 1.4 g (85%).

^{1}H NMR (500 MHz, $CDCl_3$) δ 8.03 (s, 2H), 7.93 (d, *J* = 8.1 Hz, 2H), 7.58 (s, 2H), 7.48 (d, *J* = 8.0 Hz, 2H), 7.27 (d, *J* = 8.1 Hz, 2H), 7.16 (d, *J* = 8.1 Hz, 2H), 3.12 – 3.00 (m, 4H), 2.03 – 1.94 (m, 4H), 1.38 (s, 12H).

^{13}C NMR (126 MHz, $CDCl_3$) δ 142.57, 140.23, 139.49, 136.52, 136.13, 134.32, 132.09, 128.19, 127.21, 126.15, 125.47, 125.43, 124.82, 90.47, 37.69, 33.32, 30.39, 23.29.

Hi-res Mass Spectrum [M+H$^+$] $C_{38}H_{33}Br_4S_2$ Cald: 872.8720 Found: 872.8712

6,6'-(9,10-di(9H-fluoren-9-ylidene)-9,10-dihydroanthracene-2,6-diyl)bis(4,4-dimethylthiochromane) (bFA-t)

Compound **5** (0.5 g, 0.57 mmol) and 5,5-dimethyl-5H-dibenzo[b,d]stannole (0.38 g, 1.25 mmol) was added into a pre-dried schlenk flask (50 mL). This flask was degassed and filled by argon via pump-refill cycles 3 times, then 25 mL dry DMF was added. The mixture was stirred under argon for 15 mins, then cesium fluoride (CsF) (0.19g, 1.25 mmol) and $Pd(tBu_3P)_2$ (32 mg, 0.06 mmol) were added. The flask was heated up to 100 °C for 24 h, then cooled to room temperature. The mixture was diluted with 100 mL DCM, and then washed with water (3×100 mL). The organic phase was collected, and dried over Sodium Sulfate anhydrous. The solvent was removed by rotor vapor, and the residue solid was further purified by silica gel column (Hexanes : DCM = 5:1) to obtain pure product as light yellow solid 0.32g (65%).

^{1}H NMR (500 MHz, $CDCl_3$) δ 8.24 (d, *J* = 7.9 Hz, 2H), 8.21 (2, *J* = 1.5 Hz, 2H), 8.16 (d, *J* = 8.0 Hz, 2H), 8.04 (d, *J* = 8.0 Hz, 2H), 7.71 (t, *J* = 7.5 Hz, 4H), 7.63 (d, *J* = 1.7 Hz, 2H), 7.51 (dd, *J* = 8.0, 1.7 Hz, 2H), 7.31 (m, 6H), 7.15 (d, *J* = 8.2 Hz, 2H), 7.13 – 7.05 (m, 4H), 3.10 – 3.02 (m, 4H), 1.99 (m, 4H), 1.38 (s, 6H), 1.34 (s, 6H).

^{13}C NMR (126 MHz, $CDCl_3$) δ 142.61, 141.17, 141.00, 140.60, 139.10, 138.60, 138.49, 137.89, 136.36, 131.84, 131.07, 129.39, 128.58, 128.55, 127.26, 126.38, 126.33, 125.91, 125.76, 125.28, 124.73, 124.67, 119.75, 119.63, 37.79, 33.36, 30.43, 30.35, 23.31.

Hi-res Mass Spectrum [M+H$^+$] $C_{62}H_{49}S_2$ Cald: 857.3276 Found: 857.3281

6,6'-(9,10-di(9H-fluoren-9-yl)anthracene-2,6-diyl)bis(4,4-dimethylthiochromane) (bFAH-t)

Compound **5** (0.5 g, 0.57 mmol) and 5,5-dimethyl-5H-dibenzo[b,d]stannole (0.38 g, 1.25 mmol) was added into a pre-dried microwave tube (10-20 mL) then 10 mL dry DMF was added. This flask was bubbled with nitrogen for 20 min, then cesium fluoride (CsF) (0.19g, 1.25 mmol) and $Pd(tBu_3P)_2$ (32 mg, 0.06 mmol) were added. The tube was sealed and was heated up to 180 °C for 20 min, then cooled to room temperature. The mixture was diluted with 100 mL DCM, and then washed with water (3×100 mL). The organic phase was collected, and dried over Sodium Sulfate anhydrous. The solvent was removed by rotor vapor, and the residue solid was purified by silica gel column (Hexanes : DCM = 5:1), and recrystallized in Hexanes to obtain pure product as light yellow crystals 0.25g (51%).

^{1}H NMR (500 MHz, CD_2Cl_2) δ 8.74 (d, *J* = 9.3 Hz, 2H), 8.08 (d, *J* = 7.6 Hz, 4H), 7.70 (d, *J* = 9.0 Hz, 2H), 7.50 (t, *J* = 7.4 Hz, 4H), 7.26 (t, *J* = 7.3 Hz, 4H), 7.21 (d, *J* = 7.4 Hz, 4H), 7.09 (s, 2H), 7.03 (s, 2H), 6.98 (d, *J* = 8.1 Hz, 2H), 6.65 (d, *J* = 8.1 Hz, 2H), 6.61 (s, 2H), 3.06 – 3.00 (m, 4H), 2.01 – 1.92 (m, 4H), 1.31 (s, 12H).

^{13}C NMR (126 MHz, CD_2Cl_2) δ 149.66, 142.77, 140.40, 136.41, 136.06, 132.66, 131.95, 130.11, 128.00, 127.70, 127.18, 125.99, 125.62, 125.09, 124.93, 124.81, 123.68, 121.15, 49.81, 38.20, 33.46, 30.53, 23.45.

Hi-res Mass Spectrum [M+H$^+$] $C_{62}H_{51}S_2$ Cald: 859.3432 Found: 859.3446

S3. Crystal structure

Single crystal X-ray diffraction. Data for all compounds was collected on an Agilent SuperNova diffractometer using mirror-monochromated Cu Kα radiation. Data collection, integration, scaling (ABSPACK) and absorption correction (face-indexed Gaussian integration or numeric analytical methods) were performed in CrysAlisPro. Structure solution was performed using ShelXT. Subsequent refinement was performed by full-matrix least-squares on F^2 in ShelXL. Olex2 was used for viewing and to prepare CIF files. ORTEP graphics were prepared in CrystalMaker. Thermal ellipsoids are rendered at the 50% probability level.

bFA was crystallized by layering hexanes on a concentrated solution in chloroform to afford bunches of colorless needles. Part of a crystal (.20 x .04 x .04 mm) was separated carefully, mounted with STP oil treatment, and cooled to 100 K on the diffractometer. Complete data were collected to 0.83 Å. 8719 reflections were collected (4969 unique, 4368 observed) with R(int) 2.4% and R(sigma) 3.6% after Gaussian absorption and beam profile correction (Tmax .981, Tmin .947). The space group was unambiguously $P2_1/n$ and the structure solved readily using ShelXT. All carbon

atoms were found in the initial solution and subsequently refined with unrestrained anisotropic ADPs. C-H hydrogens were placed in calculated positions and refined with riding coordinates and ADPs. The final refinement (4969 data, 0 restraints, 361 parameters) converged with R_1 ($F_o > 4\sigma(F_o)$) = 4.2%, wR_2 = 10.8%, S = 1.03. The largest Fourier features were 0.27 and -0.23 $e^- A^{-3}$.

bFT was crystallized by layering hexanes on a concentrated solution in dichloromethane to afford irregular colorless crystals. Part of a crystal (.14 x .12 x .08 mm) was separated carefully, mounted with STP oil treatment, and cooled to 100 K on the diffractometer. Complete data were collected to 0.83 Å. 8674 reflections were collected (5523 unique, 4621 observed) with R(int) 2.9% and R(sigma) 4.9% after Gaussian absorption and beam profile correction (Tmax .968, Tmin .946). The space group was unambiguously $P2_1/n$ and the structure solved readily using ShelXT. All carbon atoms were found in the initial solution and subsequently refined with unrestrained anisotropic ADPs. C-H hydrogens were placed in calculated positions and refined with riding coordinates and ADPs. The final refinement (5523 data, 0 restraints, 397 parameters) converged with R_1 ($F_o > 4\sigma(F_o)$) = 4.4%, wR_2 = 11.2%, S = 1.03. The largest Fourier features were 0.22 and -0.26 $e^- A^{-3}$.

bFP was crystalized from slow evaporation of a chloroform solution to afford large, pale yellow rectangular rods. Part of a crystal (.43 x .14 x .09 mm) was separated carefully, mounted with STP oil treatment, and cooled to 100 K on the diffractometer. Complete data (99.6%) were collected to 0.815 Å. 47924 reflections were collected (6423 unique, 6166 observed) with R(int) 5.9% and R(sigma) 2.7% after analytical absorption correction (Tmax .873, Tmin .658). The space group was assigned as C2/c based on the systematic absences. The structure solved readily in ShelXT with 1 molecule in the asymmetric unit. All non-H atoms were located in Fourier maps and refined anisotropically with no restraints. A chloroform molecule was located on a twofold axis, this special position disorder did not require any special treatment once the correct positions of all atoms had been identified. C-H hydrogens were placed in calculated positions and refined with riding coordinates and ADPs. The final refinement (6423 data, 0 restraints, 469 parameters) converged with R_1 ($F_o > 4\sigma(F_o)$) = 5.3%, wR_2 = 14.1%, S = 1.02. The largest Fourier features were 0.52 and -0.61 $e^- A^{-3}$.

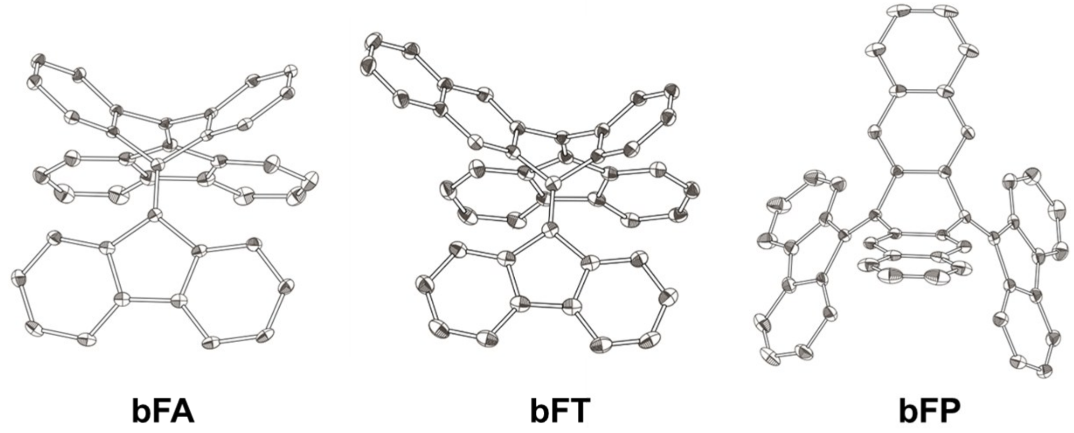

Figure S1. Molecular structure of **bFA, bFT**, and **bFP.**

Table S1. X-ray Diffraction Data of **bFA, bFT** and **bFP**

**Compound**	**bFA**	**bFT**	**bFP•0.5 $CHCl_3$**
**Formula**	$C_{40}H_{24}$	$C_{44}H_{26}$	$C_{48.5}H_{28.5}Cl_{1.5}$
**MW**	504.59	554.65	664.39
**Space group**	$P2_1/n$	$P2_1/n$	C2/c
a **(Å)**	9.73779(16)	11.6946(5)	17.38224(13)
b **(Å)**	17.0425(3)	15.9924(6)	15.79468(12)
c **(Å)**	15.4561(3)	15.5144(6)	24.01834(18)
α **(°)**	90	90	90
β **(°)**	91.0108(14)	97.492(4)	92.2024(7)
γ **(°)**	90	90	90
**V ($Å^3$)**	2564.63(8)	2876.8(2)	6589.29(9)
**Z**	4	4	8
**ρ_{calc} (g cm^{-3})**	1.307	1.281	1.339
**T (K)**	100	100	100
**λ (Å)**	1.54184	1.54184	1.54184
**$2\theta_{min}$, $2\theta_{max}$**	7.7, 146	8, 146	8.33, 143.17
**Nref**	8719	8674	47924
**R(int), R(σ)**	0.0235, 0.0365	.0286, .0491	.0587, .0269
**μ(mm^{-1})**	0.566	0.552	1.668
**Size (mm)**	.21 x .04 x .04	.14 x .12 x .08	.43 x .14 x .09
**T_{max}, T_{min}**	.981, .937	.968, .946	.873, .658
**Data**	4969	5523	6423
**Restraints**	0	0	0
**Parameters**	361	397	469
**R_1(obs)**	0.0421	0.0439	0.0531
**wR_2(all)**	0.1076	0.1124	0.1415
**S**	1.028	1.029	1.023
**Peak, hole (e^- $Å^{-3}$)**	0.27, -0.23	0.26, -0.22	0.52, -0.61

S4. Supporting Data

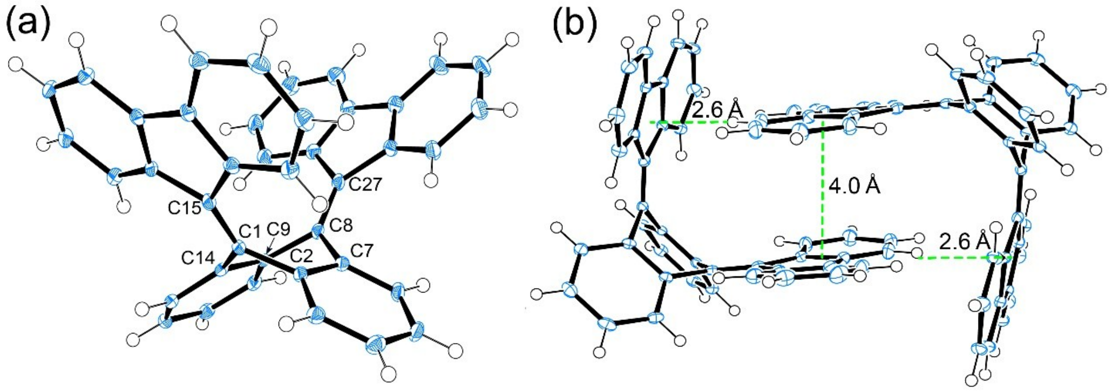

Figure S2. (a) ORTEP plots of difluorenylidene-anthraquinoid compound (**bFA**) (ellipsoid probability: 50%); (b) Dimer structure of **bFA** in crystal packing.

The acene backbones also display a typical quinoidal structure，C1-C15 and C8-C28 have double bond character (~1.35 Å) while C1-C2, C1-C14, C8-C7, C8-C9 have single bond character (~1.49 Å)

Photophysical properties of difluorenylidene dihydroacenes:

UV-visible absorption data were acquired on a Varian Cary 5000 UV-Vis/NIR spectrophotometer. The fluorescence data and lifetimes were measured using an Horiba Fluorolog-3 spectrofluorometer equipped with a 388 nm nanoLED and a FluoroHub R-928 detector.

Absolute quantum yields were measured on the HORIBA Fluorolog-3 using a pre-calibrated Quanta-φ integrating sphere. Light from the sample compartment is directed into the sphere via a fiber-optic cable and the F-3000 Fiber-Optic Adapter, and then returned to the sample compartment (and to the emission monochromator) via a second fiber-optic cable and the F-3000.

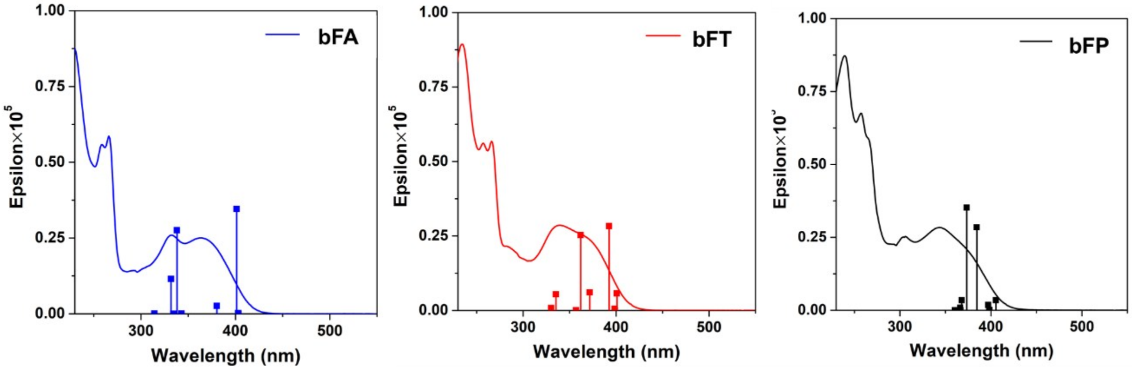

Figure S3. UV-Vis spectra of difluorenylidene dihydroacenes in THF solution (1×10^{-5}**M**). Droplines are TD-DFT calculation results (with B3LYP/6-31G** method).

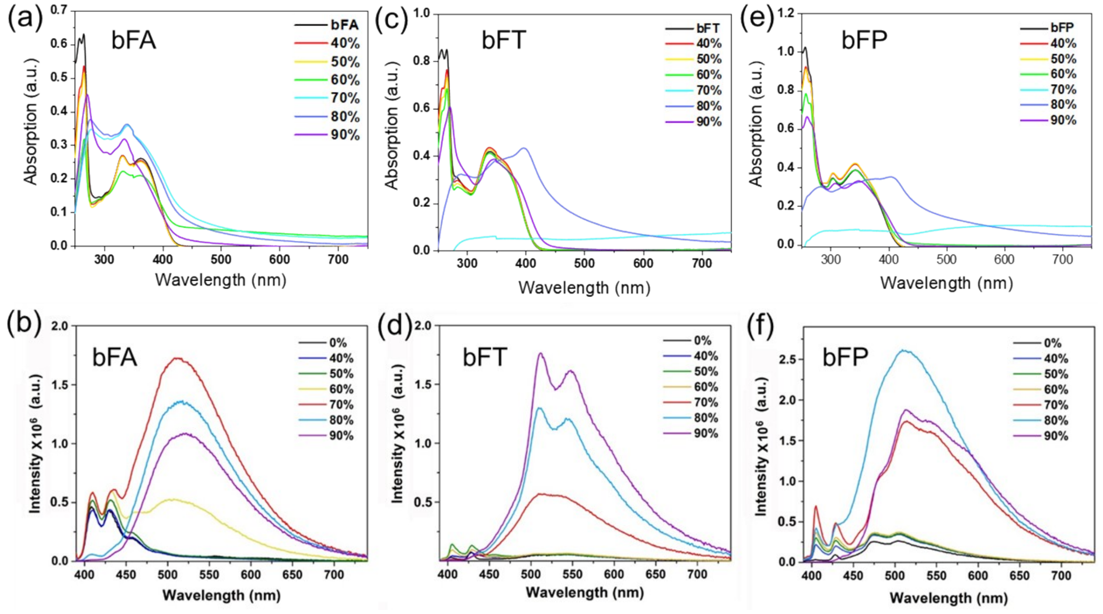

Figure S4. UV-Vis (a, c, e) and Fluorescent (b, d, f) spectra of **bFA**, **bFT** and **bFP** in their solutions of THF/water with different volume fraction of water.

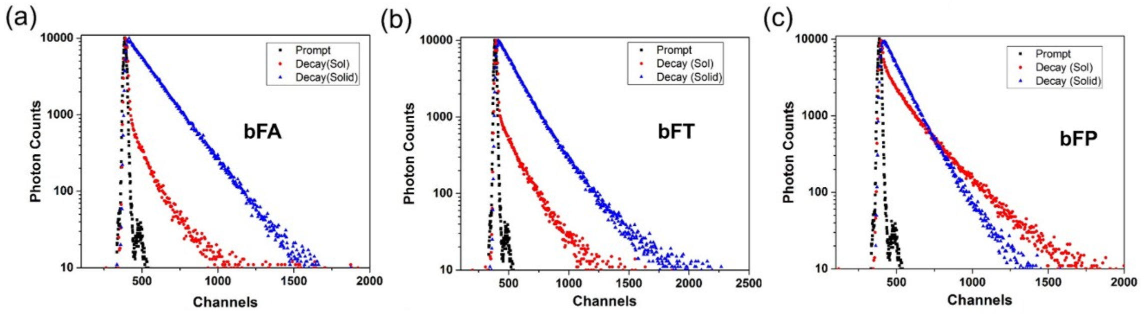

Figure S5. Fluorescence decay trace of **bFA**, **bFT** and **bFP** in the solution of THF (red plots) and thin film (blue plots).

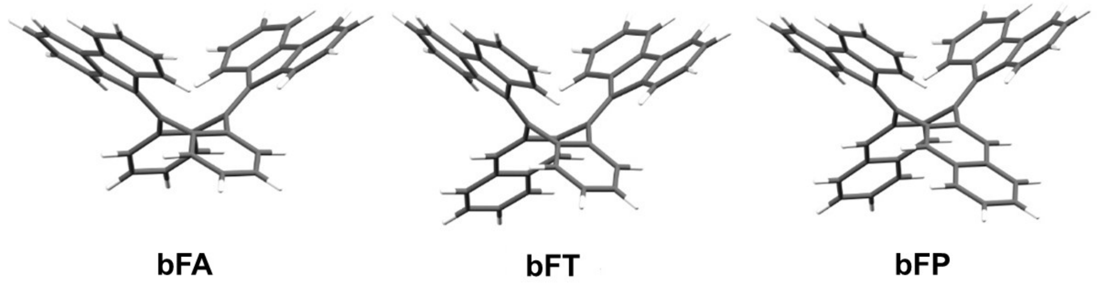

Figure S6. Optimized structure of Difluoreno-acenequinones at DFT (B3LYP/6-31G**) level.

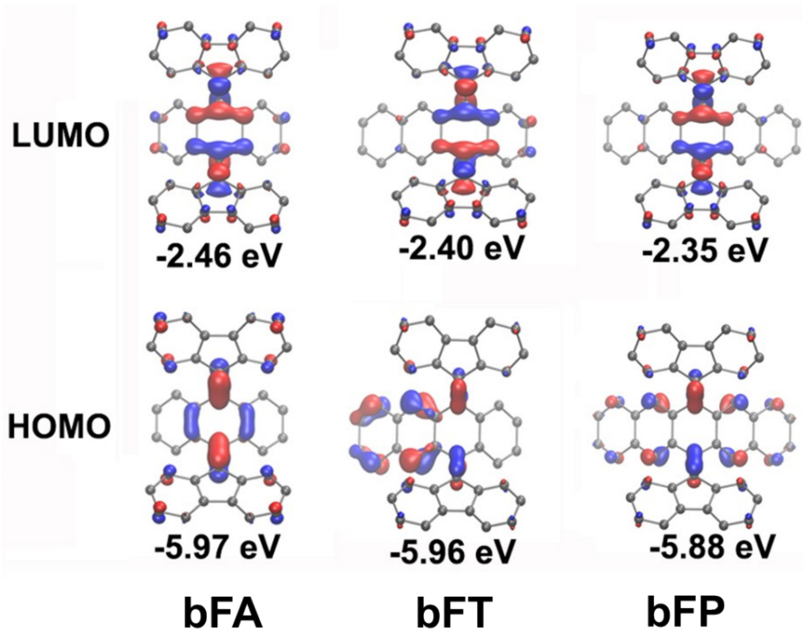

Figure S7. Orbital diagram of difluorenylidene dihydroacenes by DFT calculation at B3PW91/6-311+G* level of theory.

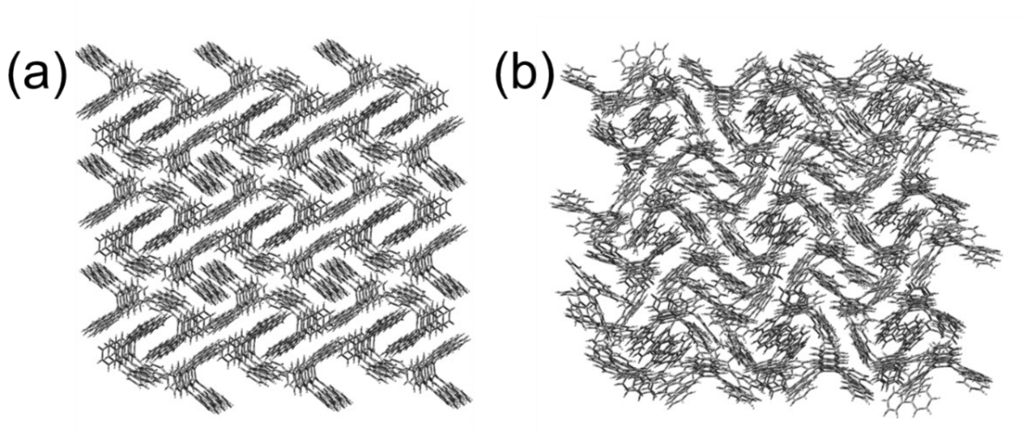

Figure S8. Molecular cluster cut from X-ray structure of **bFA (a)** and **bFT (b)** for QM/MM calculation model.

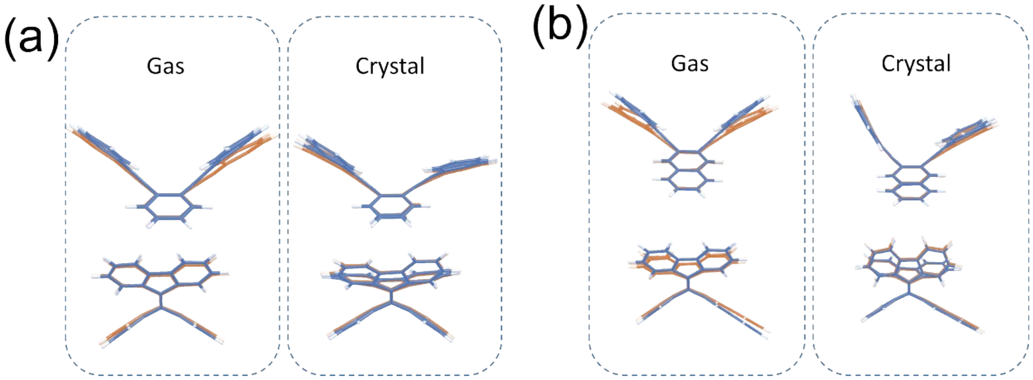

Figure S9. Optimized structure of ground state (s_0, blue) and excited state (s_1, orange) of **bFA** (a) and **bFT** (b) at gas state and crystal with B3LYP/6-31G** and ONIOM (B3LYP/6-31G**: UFF) method, respectively.

Table S2. Dihedral angles between fluorenylidene and benzene/naphthalene of ground state (S_0) and excited state (S_1) of **bFA**, **bFT**, **bFP** in the gas and crystal state.

bFA	Gas		Crystal	
	S_0	S_1	S_0	S_1
ϕ_{fluo}	109.21°	117.07°	140.71°	142.89°
ϕ_{acene}	111.25°	116.11°	113.66°	115.92°
bFT	Gas		Crystal	
	S_0	S_1	S_0	S_1
ϕ_{fluo}	105.80°	123.40°	92.32°	90.14°
ϕ_{acene}	110.82°	116.52°	118.00°	120.20°
bFP	Gas		Crystal	
	S_0	S_1	S_0	S_1
ϕ_{fluo}	97.92°	114.89°	82.33°	83.94°
ϕ_{acene}	111.07°	117.30°	116.03°	116.54°

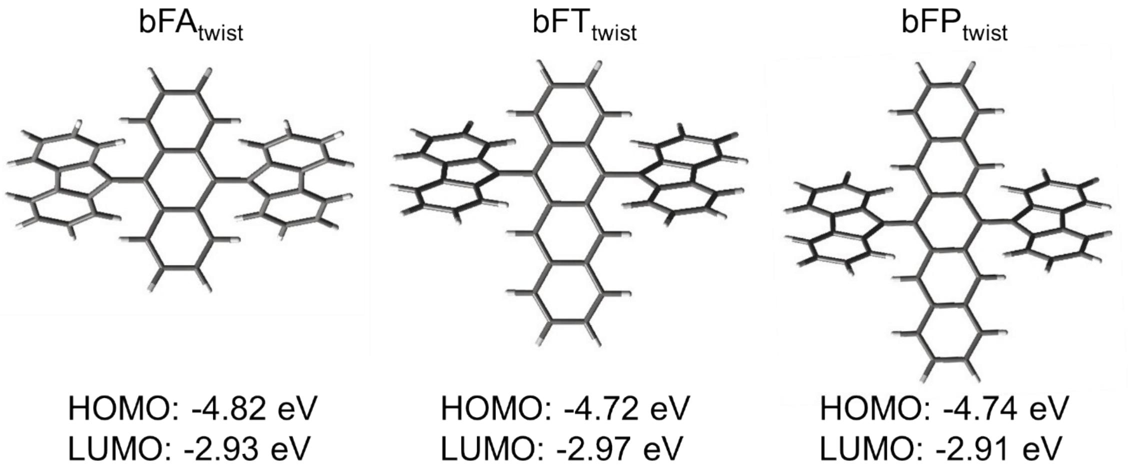

Figure S10. Optimized structure (left) and energy level of HOMO and LUMO (right) of **bFA$_{twist}$**, **bFT$_{twist}$** and **bFP$_{twist}$** by DFT calculation at UB3LYP/6-31G** level of theory.

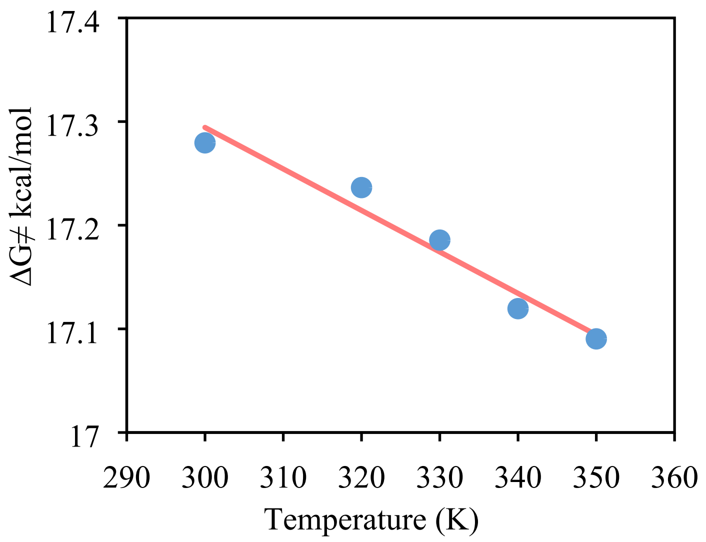

Figure S11. Plots of $\Delta G^{\neq}$ versus temperature (K) of **bFA-t**. The value of $\Delta G^{\neq}$ are obtained from the D-NMR data which was simulated with TopSpin 3.5.

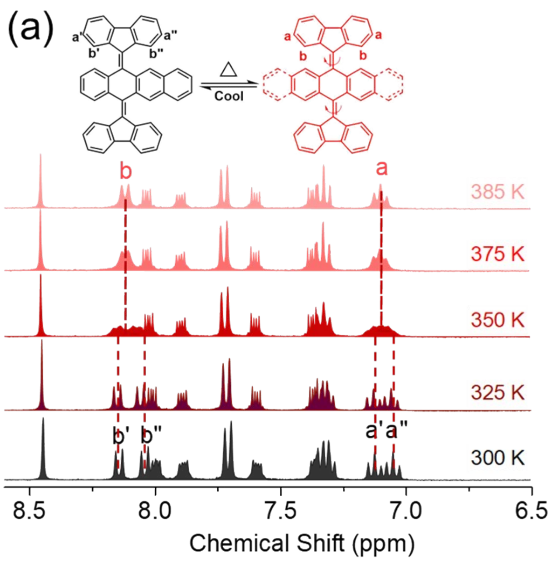

Figure S12. (a) VT-^{1}H NMR of **bFT** in tetrachloroethane-d2; (b) VT-^{1}H NMR of **bFP** in DMSO-d6

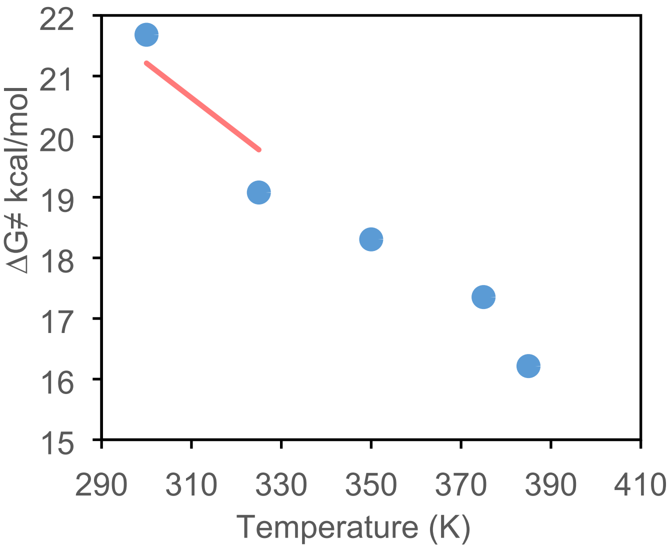

Figure S13. Plots of ΔG$^{\neq}$ versus temperature (K) of **bFT**. The value of ΔG$^{\neq}$ are obtained from the DNMR data which was simulated by TopSpin 3.5.

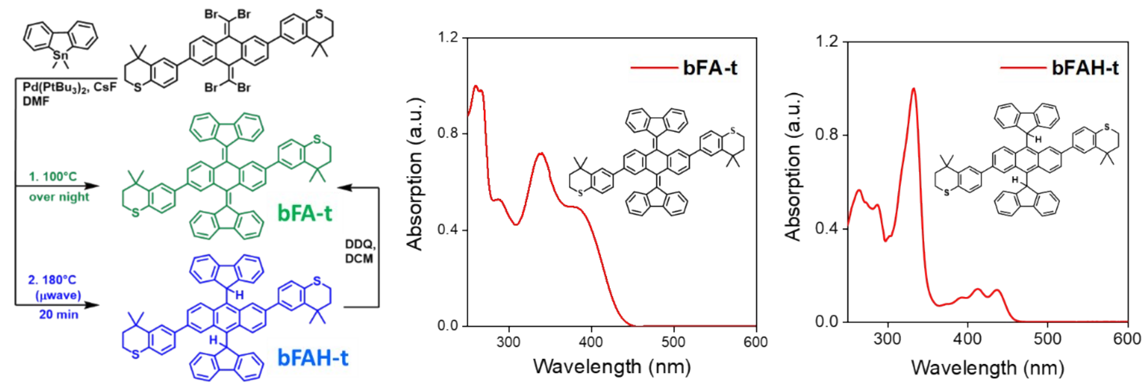

Figure S14. Normalized UV-Vis Spectra of **bFA-t** and **bFAH-t** in their DCM solution (1×10^{-5}M).

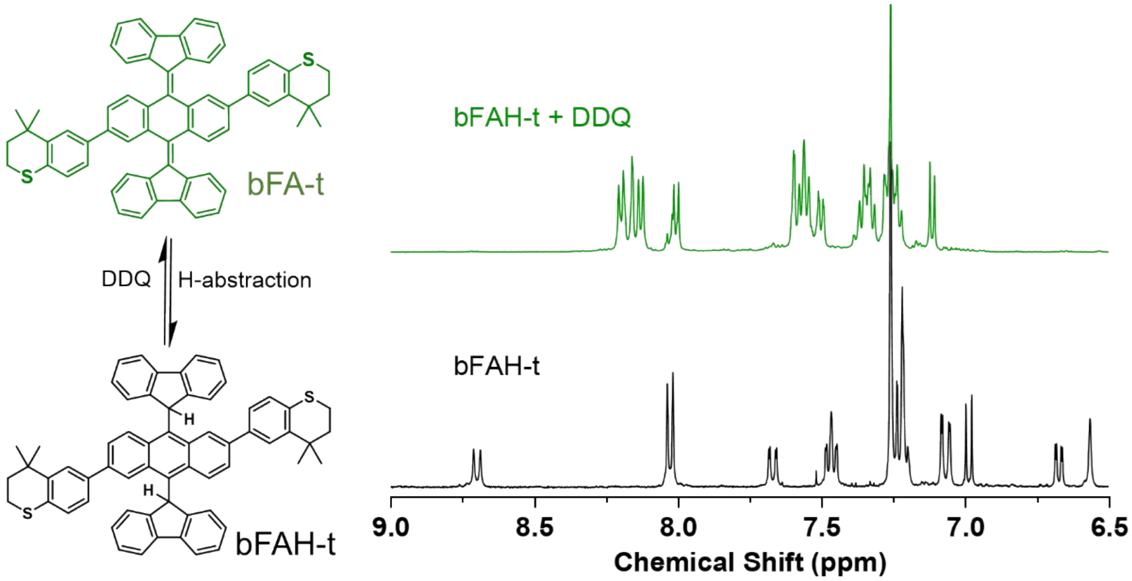

Figure S15. Illustration of reversible conversion between **bFA-t** and **bFAH-t** via H-abstraction and DDQ oxidation.

Electrochemical Properties

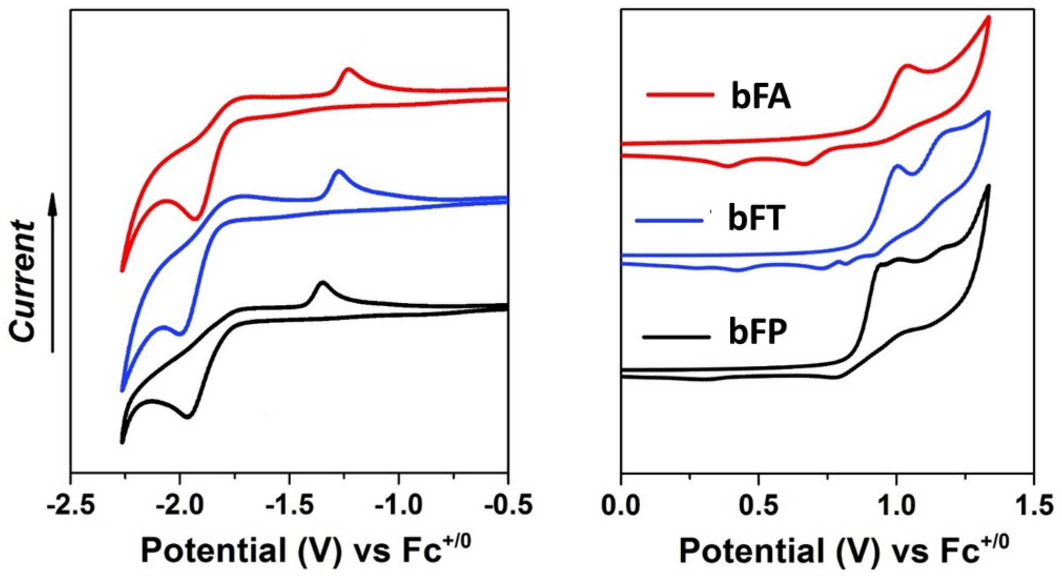

Figure S16. Cyclic Voltammetry of **bFA**, **bFT** and **bFP** in DCM solution with 0.1M $Bu_4N[PF_6]$ (1×10^{-3} mol L^{-1}; vs. $Fc^{0/+}$, ν = 100 mV s^{-1}).

The electrochemical reactivity of difluorenylidene dihydroacenes were studied by cyclic voltammetry and spectro-electrochemistry method, respectively. Cyclic voltammetry of these compounds were measured in their dichloromethane (DCM) solutions with three-electrode system (Pt disk electrode as working electrode, Pt wire as counter electrode and Ag/AgCl electrode as reference electrode), and tetrabutylammonium hexafluorophosphate ($Bu_4N[PF_6]$) as supporting electrolyte. As shown in Figure S16, all these compounds exhibit quasi-reversible redox peaks on the reduction region with redox potential at ca. -1.8 V vs $Fc^{+/0}$ and a partial recovery peak at ca. -1.3 V. On the aspect of oxidation region, the on-set value of first oxidation potential slightly change from ca. 0.8 V vs. $Fc^{+/0}$, and irreversible oxidation process can be observed for all of them.

S5. DFT Calculation Data

Coordinates of bFA optimized by Gaussian 09 (B3LYP/6-31G**)

Element	Coordinates (angstroms)			Element	Coordinates (angstroms)		
	X	Y	Z		X	Y	Z
C	0.69826100	3.20689400	2.96740600	C	4.71454100	-2.98754100	-1.89664500
C	-0.69831200	3.20689600	2.96738100	C	4.99459600	-1.62177100	-1.95350400
C	-1.39575300	2.20462900	2.29441700	C	-2.76322200	-2.55266200	-0.50982700
C	-0.70768200	1.20392400	1.59721700	C	-3.59568000	-3.44273400	-1.19444200
C	0.70768300	1.20393000	1.59722900	C	-4.71490600	-2.98732300	-1.89650300
C	1.39572600	2.20462700	2.29446300	C	-4.99480400	-1.62152200	-1.95340800
C	-1.36925600	-0.00002700	1.01098300	C	-4.99457100	1.62181600	-1.95346800
C	-0.70769200	-1.20396200	1.59726600	C	-4.71448800	2.98758000	-1.89658600
C	0.70767400	-1.20395700	1.59723400	C	-3.59520900	3.44285100	-1.19451900
C	1.36923600	-0.00001800	1.01096000	C	-2.76288700	2.55268000	-0.50986400
C	-1.39572100	-2.20464300	2.29453900	H	1.24467600	3.97099300	3.51219900
C	-0.69824600	-3.20689300	2.96750200	H	-1.24474200	3.97100100	3.51215200
C	0.69832400	-3.20689200	2.96746000	H	-2.48004900	2.18326600	2.32252900
C	1.39575300	-2.20463700	2.29446000	H	2.48002100	2.18326700	2.32262100
C	2.37161700	-0.00003300	0.08603200	H	-2.48001400	-2.18328000	2.32272300
C	-2.37163900	-0.00000700	0.08605900	H	-1.24465400	-3.97096700	3.51233700
C	3.05301400	-1.18463200	-0.52169900	H	1.24476400	-3.97097200	3.51225400
C	4.15721000	-0.73140600	-1.28456800	H	2.48004900	-2.18327100	2.32256500
C	4.15727900	0.73124700	-1.28452600	H	5.84233500	1.25782100	-2.52734900
C	3.05312300	1.18453400	-0.52163300	H	5.35508300	3.69475000	-2.41537800
C	-3.05313400	-1.18455100	-0.52166700	H	3.36169100	4.50324400	-1.18182700
C	-4.15729900	-0.73122800	-1.28452800	H	1.89542000	2.93766400	0.00724200
C	-4.15720400	0.73142400	-1.28454400	H	1.89513700	-2.93768000	0.00707200
C	-3.05300000	1.18461300	-0.52166600	H	3.36123000	-4.50332800	-1.18213100
C	4.99476900	1.62157200	-1.95338400	H	5.35468300	-3.69495200	-2.41565600
C	4.71485700	2.98736800	-1.89643000	H	5.84218800	-1.25806700	-2.52746100
C	3.59562800	3.44274300	-1.19435100	H	-1.89546100	-2.93772000	0.00715600
C	2.76318900	2.55263800	-0.50975200	H	-3.36175800	-4.50323900	-1.18194900
C	2.76293800	-2.55270900	-0.50990700	H	-5.35514700	-3.69468000	-2.41546500
C	3.59527700	-3.44285000	-1.19457900	H	-5.84236700	-1.25774300	-2.52735900
H	-3.36114000	4.50332300	-1.18204600	H	-5.84217000	1.25813700	-2.52743000
H	-1.89507900	2.93762500	0.00712600	H	-5.35461700	3.69501400	-2.41558200

Coordinates of bFT optimized by Gaussian 09(B3LYP/6-31G**)

Element	Coordinates (angstroms)			Element	Coordinates (angstroms)		
	X	Y	Z		X	Y	Z
C	-0.70814300	6.06637100	1.50586200	C	-2.76726900	1.52410600	-1.71369400
C	0.70817900	6.06636900	1.50583800	C	-3.59240300	2.01145600	-2.73109100
C	-1.40344100	4.89048200	1.33198800	C	-4.69280000	1.27652700	-3.18079600
C	-0.71621900	3.66163400	1.14557200	C	-4.95964600	0.01869200	-2.63944900
C	0.71623600	3.66163200	1.14554900	C	-4.93858800	-2.89575400	-1.21872200
C	1.40346800	4.89047800	1.33194200	C	-4.65463100	-4.09301400	-0.56085600
C	-1.40131700	2.42927300	0.98024000	C	-3.54961400	-4.17759000	0.29049800
C	-0.71913100	1.24609800	0.78987200	C	-2.73525800	-3.06646700	0.52712500
C	0.71912800	1.24609600	0.78985600	H	-1.24479900	7.00011100	1.64522800
C	1.40132500	2.42926900	0.98019700	H	1.24484300	7.00010700	1.64518700
C	-1.36969400	-0.09747400	0.77868300	H	-2.49022300	4.88696700	1.33356800
C	-0.70771400	-0.91609200	1.83825300	H	2.49025000	4.88696000	1.33348800
C	0.70772100	-0.91609800	1.83823900	H	-2.48651400	2.42468700	1.01644100
C	1.36968700	-0.09747800	0.77866300	H	2.48652300	2.42467900	1.01636500
C	-1.39605000	-1.50180200	2.90812300	H	-2.48045400	-1.47109900	2.92226700
C	-0.69842500	-2.10014600	3.95623000	H	-1.24447500	-2.54329400	4.78367700
C	0.69846700	-2.10016100	3.95621100	H	1.24453100	-2.54332100	4.78364200
C	1.39607500	-1.50182800	2.90808600	H	2.48048000	-1.47114400	2.92220200
C	2.36303700	-0.50916300	-0.06008200	H	5.77520500	-2.83269000	-1.90874100
C	-2.36305100	-0.50916100	-0.06006100	H	5.28065900	-4.96499700	-0.72611000

C	3.04335200	0.28214500	-1.13231800	H	3.31275600	-5.12069200	0.77425500
C	4.12828100	-0.47635900	-1.63664800	H	1.87830100	-3.17295100	1.17734600
C	4.11920200	-1.79098400	-0.99573400	H	1.91527200	2.11181700	-1.40198100
C	3.02891500	-1.84788200	-0.09334900	H	3.36820900	2.97533700	-3.17858200
C	-3.04336300	0.28214300	-1.13230600	H	5.32828600	1.67575800	-3.96588800
C	-4.12831600	-0.47634800	-1.63660600	H	5.79219900	-0.57317400	-3.00907100
C	-4.11922400	-1.79097800	-0.99570600	H	-1.91526100	2.11179500	-1.40201600
C	-3.02890900	-1.84789200	-0.09335700	H	-3.36822900	2.97532400	-3.17858300
C	4.93856100	-2.89576300	-1.21875000	H	-5.32834900	1.67577200	-3.96582900
C	4.65462900	-4.09300800	-0.56084600	H	-5.79226600	-0.57315000	-3.00899100
C	3.54964400	-4.17756500	0.29055400	H	-5.77525100	-2.83266900	-1.90868800
C	2.73529400	-3.06643800	0.52718200	H	-5.28066300	-4.96500100	-0.72612200
C	2.76726500	1.52411900	-1.71368600	H	-3.31270500	-5.12072900	0.77416600
C	3.59237900	2.01146300	-2.73110100	H	-1.87824200	-3.17299600	1.17725400
C	4.69275100	1.27651700	-3.18084200				
C	4.95959300	0.01867500	-2.63950900				

Coordinates of bFP optimized by Gaussian 09(B3LYP/6-31G)**

Element	Coordinates (angstroms)			Element	Coordinates (angstroms)		
	X	Y	Z		X	Y	Z
C	-0.71922000	1.19992300	0.94807200	C	-4.67636400	2.98678100	-2.61152600
C	0.71907600	1.20002800	0.94782200	C	-4.94996500	1.62042100	-2.67801800
C	1.37211100	0.00005200	0.34625100	C	2.75524800	-2.55501500	-1.18175100
C	0.71920400	-1.19998400	0.94784400	C	3.57480300	-3.44399600	-1.88272800
C	-0.71909300	-1.20003700	0.94808200	C	4.67586800	-2.98680200	-2.61207000
C	-1.37230000	-0.00009300	0.34660800	C	4.94925500	-1.62041300	-2.67891100
C	-1.40107300	2.17835000	1.64122500	C	4.94905700	1.62096300	-2.67888400
C	-0.71627300	3.21224800	2.33140000	C	4.67550400	2.98731900	-2.61201500
C	0.71637500	3.21241300	2.33104600	C	3.57438700	3.44436600	-1.88266100
C	1.40106500	2.17864200	1.64057600	C	2.75494000	2.55527200	-1.18169900
C	-1.40345700	4.23451700	3.03882400	H	-2.48622100	2.15828300	1.67231300
C	-0.70809500	5.21445700	3.71120000	H	2.48623500	2.15879900	1.67114100
C	0.70842200	5.21462100	3.71084700	H	-2.49022900	4.23068400	3.03893900
C	1.40367600	4.23484000	3.03812700	H	-1.24468200	5.99206900	4.24662700
C	1.40129400	-2.17850500	1.64062900	H	1.24509500	5.99235700	4.24600700
C	0.71671200	-3.21233900	2.33111300	H	2.49044800	4.23125000	3.03771000
C	-0.71593500	-3.21233900	2.33144500	H	2.48646200	-2.15854500	1.67120000
C	-1.40084400	-2.17852800	1.64124600	H	-2.48599500	-2.15858500	1.67231900
C	1.40411800	-4.23467600	3.03822300	H	2.49089000	-4.23096300	3.03782300
C	0.70896500	-5.21452800	3.71094300	H	1.24571800	-5.99219600	4.24612400
C	-0.70755200	-5.21452800	3.71127200	H	-1.24405800	-5.99219600	4.24669900
C	-1.40301500	-4.23467600	3.03887100	H	-2.48978700	-4.23096600	3.03896700
C	-2.36445100	-0.00014200	-0.58898000	H	-5.78390200	-1.25565800	-3.27055800
C	2.36409700	0.00010200	-0.58943800	H	-5.30741500	-3.69393000	-3.14227800
C	-3.03748300	1.18509000	-1.20627200	H	-3.34530100	-4.50564400	-1.86063100
C	-4.12398800	0.73094800	-1.99345600	H	-1.90259400	-2.94245000	-0.64181400
C	-4.12390900	-0.73135400	-1.99349500	H	-1.90292100	2.94221000	-0.64163800
C	-3.03735500	-1.18542000	-1.20633400	H	-3.34580200	4.50531900	-1.86036100
C	3.03729300	-1.18512200	-1.20650300	H	-5.30782300	3.69346200	-3.14206300
C	4.12343100	-0.73093900	-1.99416800	H	-5.78404000	1.25514500	-3.27048700
C	4.12334200	0.73137600	-1.99415600	H	1.90327700	-2.94228900	-0.64094500
C	3.03715000	1.18541500	-1.20648200	H	3.34589600	-4.50542200	-1.86000900
C	-4.94978800	-1.62087800	-2.67810900	H	5.30722700	-3.69348700	-3.14272000
C	-4.67603500	-2.98721000	-2.61169900	H	5.78309100	-1.25512700	-3.27171100
C	-3.57451000	-3.44427500	-1.88298000	H	5.78293600	1.25578900	-3.27169200
C	-2.75488600	-2.55524600	-1.18214300	H	5.30677600	3.69409100	-3.14265400
C	-2.75516900	2.55494400	-1.18199400	H	3.34535200	4.50576300	-1.85991800
C	-3.57489200	3.44392600	-1.88277700	H	1.90292400	2.94243100	-0.64088000

Coordinates of bFA_{twist} optimized by Gaussian 09(UB3LYP/6-31G)**

Element	Coordinates (angstroms)			Element	Coordinates (angstroms)		
	X	Y	Z		X	Y	Z
C	0.69153100	-3.69102900	-0.12707200	C	5.88980800	1.90051600	-2.33473600
C	-0.69163100	-3.69101500	0.12687000	C	4.55972800	2.20027800	-2.66075900
C	-1.37039600	-2.49348000	0.19939600	C	3.50379200	1.65703200	-1.93126600
C	-0.71570700	-1.24387000	0.02703700	C	3.50366800	-1.65705400	1.93126800
C	0.71568600	-1.24388700	-0.02713400	C	4.55955900	-2.20031800	2.66081300
C	1.37033200	-2.49350400	-0.19955400	C	5.88966000	-1.90057000	2.33486400
C	-1.46884000	0.00000500	-0.00004200	C	6.17881200	-1.03146400	1.28066400
C	-0.71570400	1.24387900	-0.02714600	C	-3.50374800	-1.65690300	-1.93137500
C	0.71568800	1.24390000	0.02702900	C	-4.55967000	-2.20011700	-2.66091700
C	1.46885100	0.00000600	-0.00003900	C	-5.88974700	-1.90038100	-2.33488900
C	-1.37039800	2.49348400	-0.19953100	C	-6.17884900	-1.03134300	-1.28060700
C	-0.69163700	3.69102200	-0.12702700	C	-6.17878400	1.03137700	1.28073700
C	0.69152400	3.69104400	0.12692200	C	-5.88962800	1.90041200	2.33500700
C	1.37032900	2.49352200	0.19943000	C	-4.55953400	2.20013800	2.66097500
C	2.88854700	-0.00000100	-0.00000900	C	-3.50364900	1.65691600	1.93138400
C	-2.88855800	0.00000800	-0.00001300	H	1.22885800	-4.62628700	-0.25167000
C	3.77660700	-0.81446700	0.84225000	H	-1.22898800	-4.62626400	0.25140400
C	5.12915800	-0.48628300	0.54468000	H	-2.43521200	-2.50372000	0.38517600
C	5.12919300	0.48624800	-0.54458500	H	2.43514100	-2.50379300	-0.38534700
C	3.77666200	0.81444900	-0.84222700	H	-2.43521200	2.50371500	-0.38531600
C	-3.77662200	-0.81439300	-0.84228000	H	-1.22899500	4.62626700	-0.25158300
C	-5.12918100	-0.48622300	-0.54461600	H	1.22884900	4.62630500	0.25150400
C	-5.12915300	0.48625000	0.54469700	H	2.43513700	2.50382000	0.38522800
C	-3.77657800	0.81441000	0.84230100	H	7.20864100	0.77203800	-1.05064500
C	6.17889300	1.03141200	-1.28051500	H	6.69868600	2.33360300	-2.91567900
H	-2.48146200	-1.89446100	-2.20463300	H	4.34692000	2.86129500	-3.49571800
H	-4.34684900	-2.86104800	-3.49594000	H	2.48151300	1.89462900	-2.20451500
H	-6.69861800	-2.33344800	-2.91585700	H	2.48137200	-1.89464000	2.20446000
H	-7.20860400	-0.77196500	-1.05077300	H	4.34669700	-2.86133700	3.49575700
H	-7.20855100	0.77200500	1.05095000	H	6.69850100	-2.33367000	2.91584800
H	-6.69847000	2.33348400	2.91601200	H	7.20857600	-0.77210300	1.05084900
H	-4.34667100	2.86106800	3.49598800	H	-2.48134900	1.89446900	2.20459600

Coordinates of bFT_{twist} optimized by Gaussian 09(UB3LYP/6-31G)**

Element	Coordinates (angstroms)			Element	Coordinates (angstroms)		
	X	Y	Z		X	Y	Z
C	0.68973700	-4.20530300	0.13413400	C	4.59741500	-2.77097000	-2.58998700
C	-0.68959100	-4.20532300	-0.13423700	C	5.92363800	-2.46238400	-2.25576200
C	1.36746600	-3.00621800	0.20994300	C	6.19898000	-1.56908800	-1.21922300
C	0.71611100	-1.75771100	0.02741000	C	6.17378100	0.55380200	1.28631500
C	-0.71605600	-1.75773500	-0.02742000	C	5.87860000	1.45011400	2.31428500
C	-1.36736300	-3.00625900	-0.21000400	C	4.54580600	1.76140400	2.61901000
C	1.47619200	-0.51489300	-0.00958000	C	3.49572700	1.20411100	1.89228200
C	0.72238900	0.73404400	-0.05088300	H	2.43067000	-3.01606000	0.40414400
C	-0.72240800	0.73402000	0.05089200	H	-2.43056400	-3.01613400	-0.40421800
C	-1.47617500	-0.51493900	0.00959800	H	2.42246400	1.96945100	-0.48156500
C	1.36255200	1.95808100	-0.26327300	H	-2.42253000	1.96937300	0.48154600
C	0.69792200	3.19205000	-0.16519400	H	-1.20753000	6.56767600	0.30152800
C	-0.69802800	3.19202800	0.16516700	H	-2.42282700	4.43362400	0.59372200

C	-1.36261500	1.95803800	0.26326200	H	-7.22540200	-1.30163700	0.98395200
C	0.68931000	5.62203000	-0.17271400	H	-6.73953900	-2.90732600	2.81770900
C	-0.68950100	5.62200900	0.17264900	H	-4.39447300	-3.45017500	3.41280600
C	-1.36659700	4.43754800	0.33891600	H	-2.51462000	-2.45918000	2.16476800
C	-2.89144200	-0.51092900	-0.00086000	H	-2.47240800	1.45635500	-2.14760400
C	2.89145900	-0.51086200	0.00087300	H	-4.32598400	2.44491400	-3.43376000
C	-3.77457300	0.33167600	-0.82857100	H	-6.68310900	1.89648200	-2.89115800
C	-5.12832200	-0.00782500	-0.55476100	H	-7.20441900	0.28470500	-1.07207600
C	-5.13961400	-1.00869300	0.50772400	H	2.51462500	-2.45919500	-2.16467300
C	-3.79155600	-1.34713600	0.81055500	H	4.39447300	-3.45022500	-3.41269400
C	3.79157000	-1.34709600	-0.81051500	H	6.73954200	-2.90734400	-2.81763900
C	5.13962900	-1.00864200	-0.50770100	H	7.22541400	-1.30159600	-0.98393500
C	5.12834100	-0.00775300	0.55476500	H	7.20444000	0.28478700	1.07206300
C	3.77459200	0.33174800	0.82857800	H	6.68313900	1.89658200	2.89113200
C	-6.19896900	-1.56912100	1.21925500	H	4.32601500	2.44500600	3.43374900
C	-5.92363200	-2.46238200	2.25582400	H	2.47243400	1.45643000	2.14761500
C	-4.59741100	-2.77094900	2.59007300	C	1.36644700	4.43759000	-0.33896300
C	-3.53301700	-2.21344200	1.88452700	H	1.20730600	6.56771400	-0.30160800
C	-3.49570200	1.20403400	-1.89227700	H	2.42267700	4.43369900	-0.59377000
C	-4.54577900	1.76131700	-2.61901800	H	1.22668500	-5.13982200	0.26535900
C	-5.87857300	1.45002300	-2.31430200	H	-1.22650400	-5.13985600	-0.26550200
C	-6.17375900	0.55372000	-1.28632600	C	3.53302500	-2.21344300	-1.88445100

Coordinates of bFP$_{twist}$ optimized by Gaussian 09(UB3LYP/6-31G**)

Element	Coordinates (angstroms)			Element	Coordinates (angstroms)		
	X	Y	Z		X	Y	Z
C	0.71093376	-5.99743988	-1.31225639	C	-4.57925173	-2.45005203	2.43303898
C	-0.71095016	-5.99742347	-1.31231195	C	-5.90890429	-2.11696882	2.13685530
C	-1.40823274	-4.84589092	-1.03672332	C	-6.19328838	-1.14952327	1.17297217
C	-0.71581859	-3.63924402	-0.73105678	C	-6.19330108	1.14950898	-1.17296000
C	0.71581013	-3.63925778	-0.73100863	C	-5.90892704	2.11695348	-2.13684631
C	1.40822109	-4.84592161	-1.03661748	C	-4.57927766	2.45004357	-2.43303793
C	-1.38019587	-2.42826104	-0.47973675	C	-3.52187409	1.84131828	-1.76032241
C	-0.72420129	-1.24665323	-0.12359570	H	1.24443862	-6.91617995	-1.53702125
C	0.72420394	-1.24666116	-0.12356606	H	-1.24445873	-6.91615138	-1.53712073
C	1.38019640	-2.42828539	-0.47965949	H	-2.49472094	-4.84004562	-1.04143564
C	-1.48847559	0.00000582	-0.00000318	H	2.49470983	-4.84009960	-1.04124673
C	-0.72420023	1.24666540	0.12358882	H	-2.45530306	-2.41578357	-0.59696274
C	0.72420499	1.24667228	0.12355760	H	2.45530888	-2.41583384	-0.59683150
C	1.48848352	0.00000529	-0.00000423	H	-2.45529988	2.41579627	0.59696168
C	-1.38019375	2.42827321	0.47973304	H	2.45531100	2.41584443	0.59682251
C	-0.71581542	3.63925566	0.73105255	H	1.24444444	6.91619054	1.53701596
C	0.71581277	3.63926889	0.73100175	H	2.49471353	4.84010965	1.04123826
C	1.38019798	2.42829597	0.47965102	H	7.22174542	-0.86596470	0.96701215
C	-0.71094487	5.99743458	1.31230931	H	6.71941492	-2.60322821	2.67168258
C	0.71093852	5.99745046	1.31225163	H	4.36824548	-3.18986723	3.19964801
C	1.40822480	4.84593219	1.03661060	H	2.50057206	-2.10978789	2.00824724
C	2.89599658	0.00000159	-0.00000106	H	2.50059799	2.10978842	-2.00825835
C	-2.89598758	0.00000370	-0.00000053	H	4.36828622	3.18985294	-3.19965013
C	3.79006218	0.89563726	-0.75882853	H	6.71944826	2.60320122	-2.67166723
C	5.13998858	0.53665770	-0.49456906	H	7.22175706	0.86593771	-0.96699046
C	5.13998223	-0.53666987	0.49457858	H	-2.50057629	-2.10991754	2.00811971
C	3.79005160	-0.89564149	0.75882906	H	-4.36825288	-3.19002122	3.19949190
C	-3.79004736	-0.89568012	0.75878673	H	-6.71942497	-2.60329753	2.67159315
C	-5.13997376	-0.53668469	0.49455689	H	-7.22174013	-0.86598375	0.96698146
C	-5.13997905	0.53667728	-0.49454842	H	-7.22175018	0.86596364	-0.96696347
C	-3.79005636	0.89568065	-0.75878514	H	-6.71945355	2.60327689	-2.67158150
C	6.19329367	-1.14949946	1.17300710	H	-4.36828728	3.19001169	-3.19949402
C	5.90890111	-2.11689632	2.13693627	H	-2.50059904	2.10992177	-2.00812923
C	4.57924591	-2.44994196	2.43315223	C	-1.40822850	4.84590256	1.03672120
C	3.52185081	-1.84122091	1.76041819	H	-1.24445291	6.91616249	1.53711968
C	3.52187356	1.84121562	-1.76042189	H	-2.49471671	4.84005779	1.04143564
C	4.57927713	2.44992821	-2.43315064	C	6.19330796	1.14947935	-1.17299228
C	5.90892810	2.11687516	-2.13692515	C	-3.52185451	-1.84131881	1.76031976

bFA			bFT			bFP		
λ (nm)	Oscillator strength *f*	Assignment	λ (nm)	Oscillator strength f	Assignment	λ (nm)	Oscillator strength *f*	Assignment
403.11	f=0.0022	H-2→L(91.5)	400.97	f=0.0684	H-1→L+1(13.9)	405.58	f=0.0411	H-1→L+1 (25.4)
		H-1→L+1(7.3)			H→L(81.8)			H→L(73.6)
401.38	f=0.4152	H→L (9.9)	398.63	f=0.0065	H-3→L(80.2)	398.16	f=0.0012	H-3→L+1(36.5)
380.36	f=0.0313	H-3→L(76.4)			H-2→L+1(15.0)			H-2→L(59.7)
		H→L+1(22.7)	392.61	f=0.3399	H-1→L(98.4)	397.1	f=0.0219	H→L+1(98.1)
343.11	f=0.0008	H-2→L (7.6)	371.72	f=0.0728	H→L+1(97.2)	384.62	f=0.3409	H-1→L(97.8)
		H-1→L+1 (91.1)	362.03	f=0.3032	H-1→L+1	373.47	f=0.4225	H-1→L+1(73.0)
338.17	f=0.3302	H-3→L(21.8)			H→L(13.0)			H→L(25.1)
		H→L+1(74.5)			H-3→L(16.1)	367.97	f=0.0413	H-4→L(77.8)
331.9	f=0.1380	H-3→L+1(98.4)	356.96	f=0.0004	H-2→L+1(80.7)			H-3→L+1(8.6)
314.25	f=0.0006	H-6→L+1(2)			H-5→L+1(7.2)	366.68	f=0.0001	H-3→L(36.4)
		H-5→L(85.7)	335.31	f=0.0655	H-4→L(80.3)			H-2→L+1(61.2)
		H-4→L+1 (3.7)	330.38	f=0.0101	H-5→L(88.7)			H-4→L(14.2)
		H-2→L+3 (2.3)			H-4→L+1(9.2)	366.45	f=0.0102	H-3→L+1(52.8)
		H-1→L+2(2.6)						H-2→L(30.1)

TD-DFT data of bFA, bFT and bFP by Gaussian 09(B3LYP/6-311+G)**

S6. Characterization Data

^{1}H NMR of bFA (25ºC, $CDCl_3$)

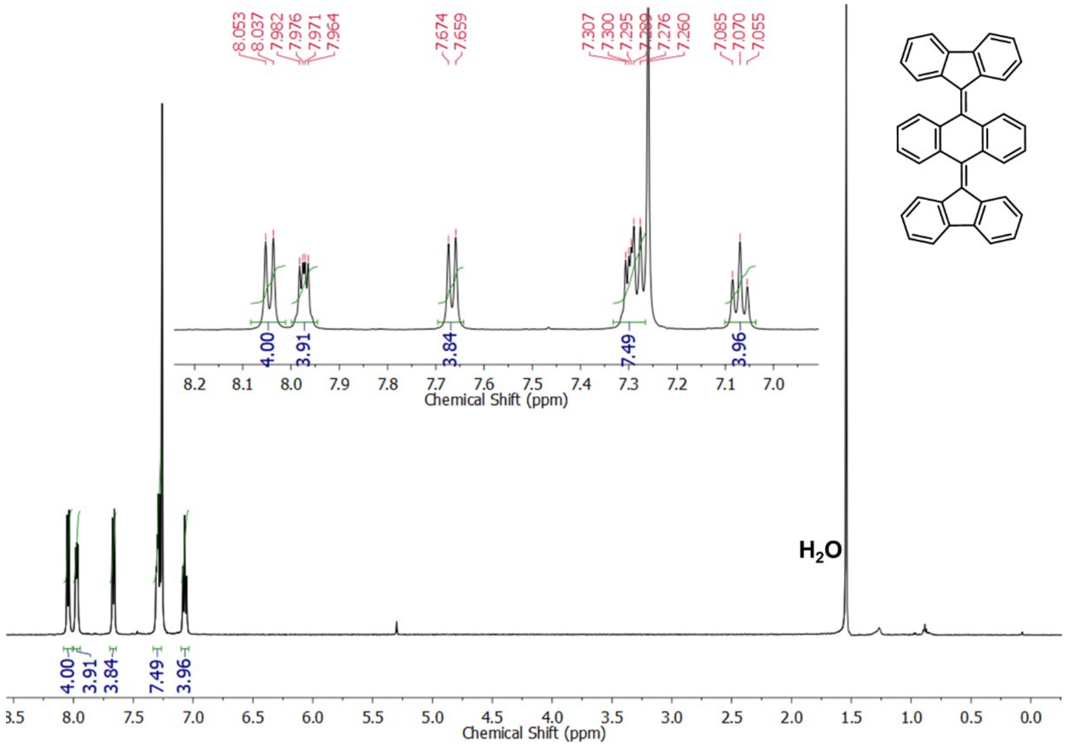

^{13}C NMR of bFA (25°C, $CDCl_3$)

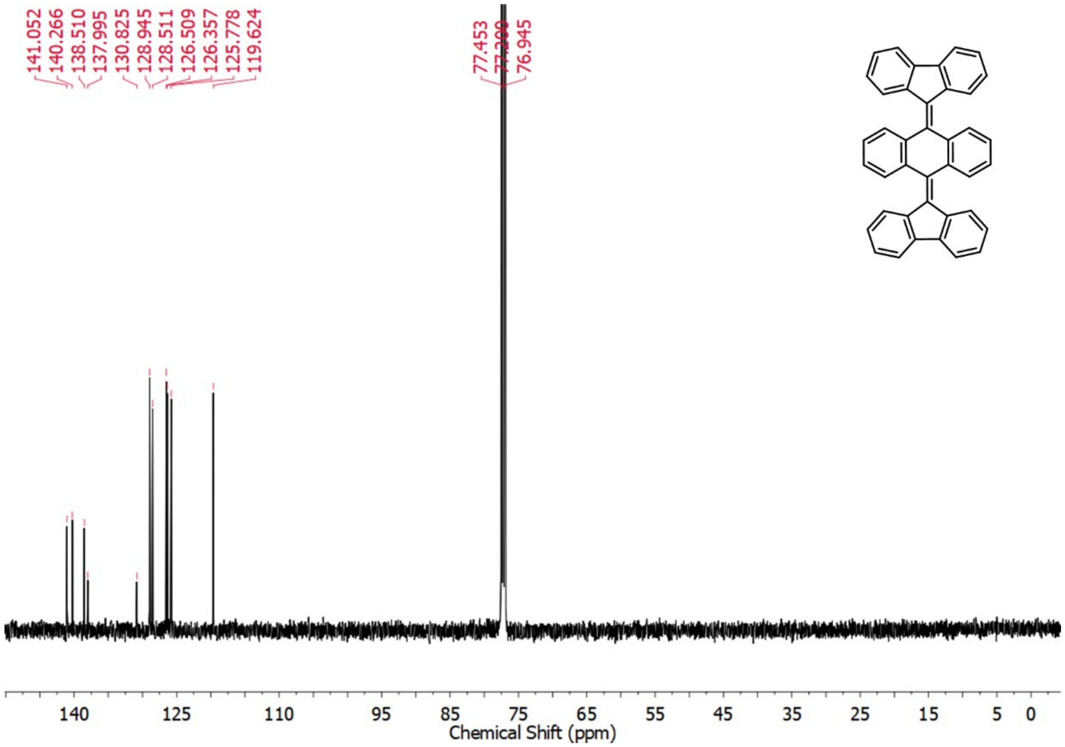

Mass Spectrum of bFA

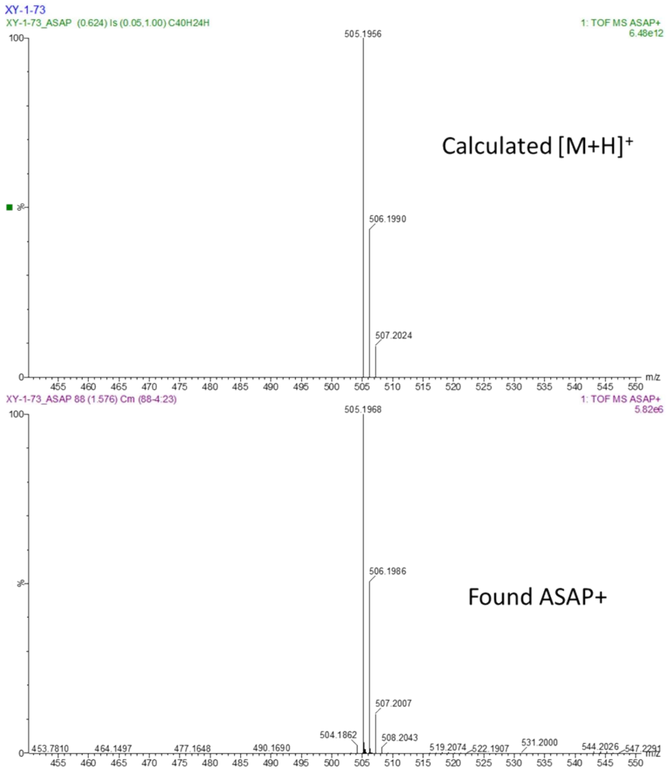

^{1}H NMR of bFT (25ºC, $CDCl_3$)

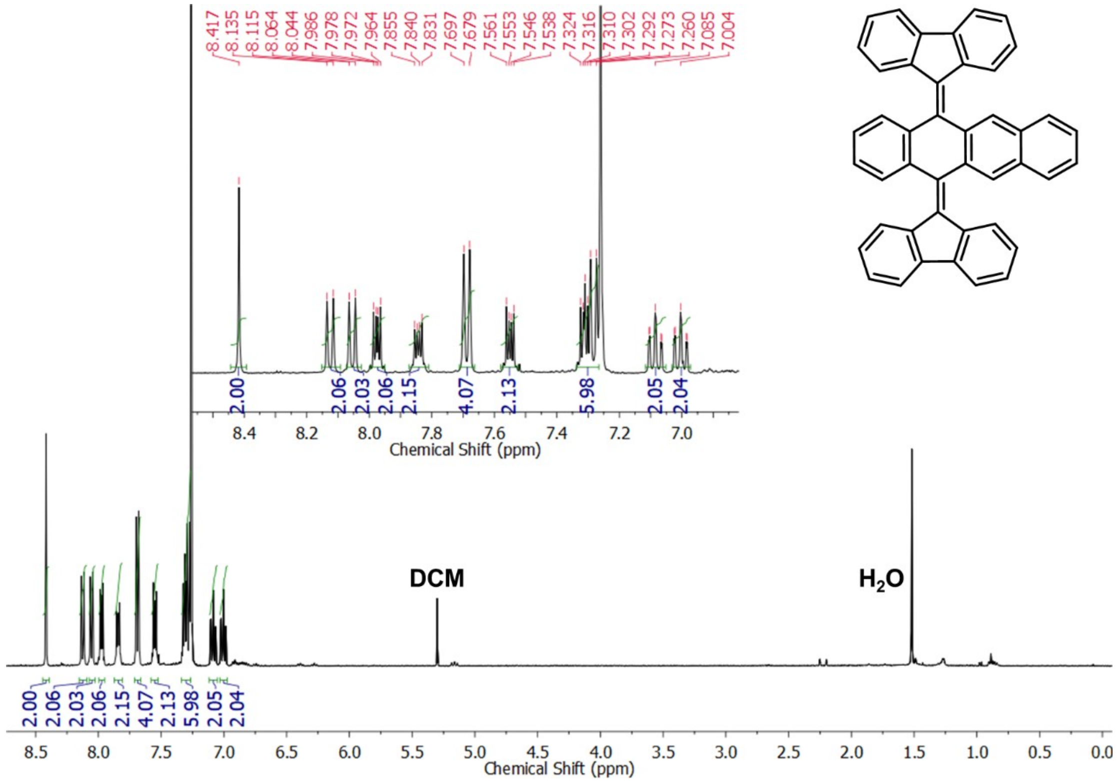

^{13}C NMR of bFT (25ºC, $CDCl_3$)

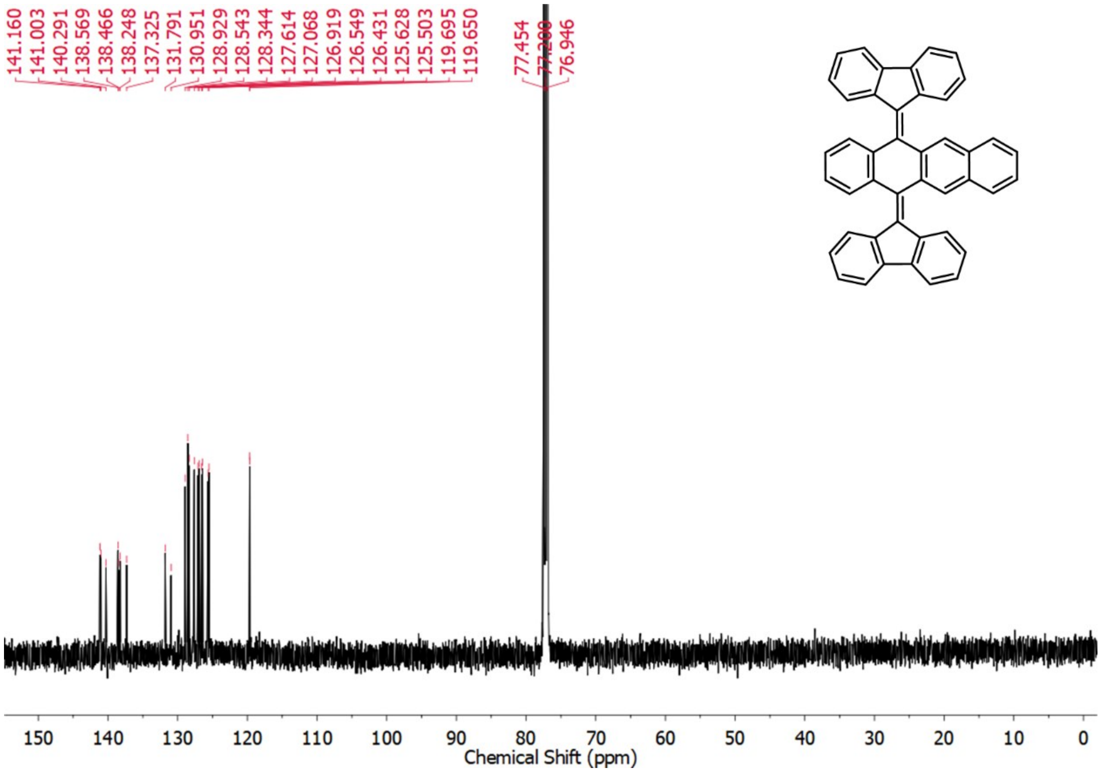

Mass Spectrum of bFT

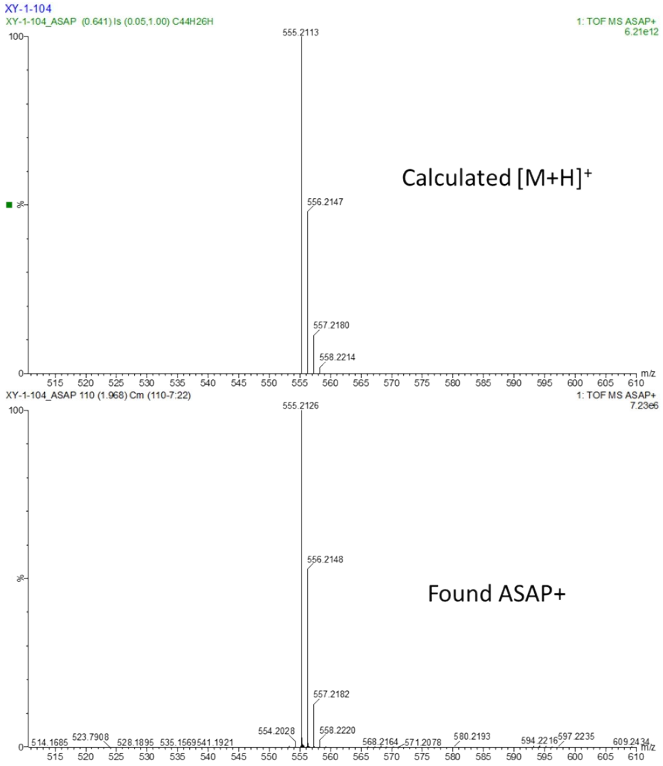

^{1}H NMR of bFP (25ºC, $CDCl_3$)

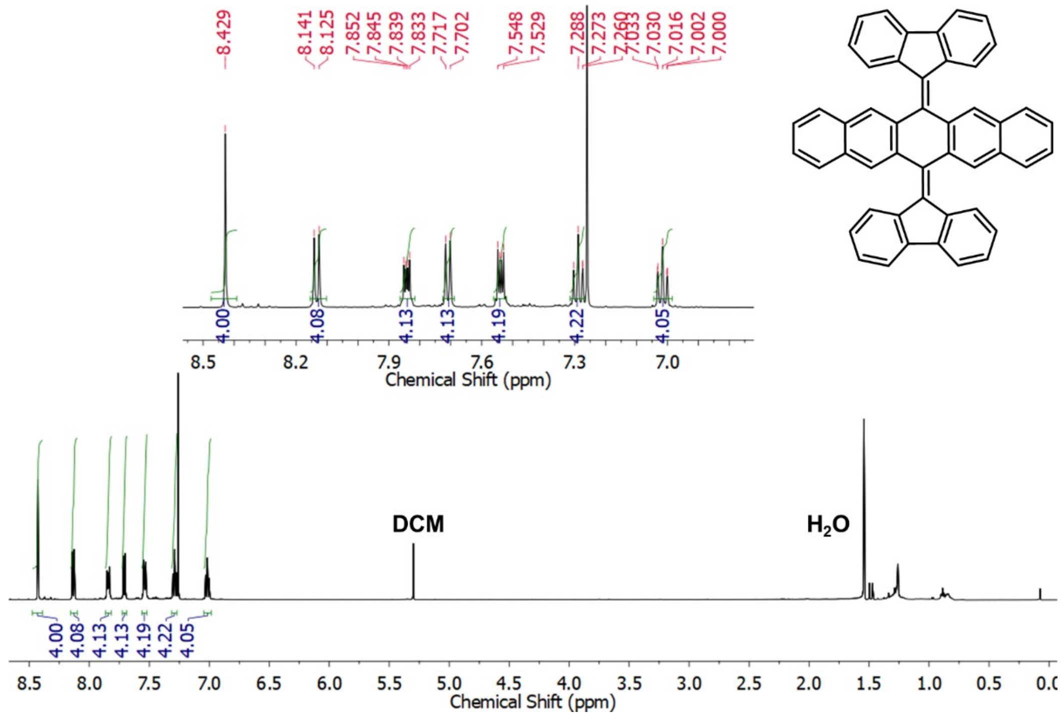

^{13}C NMR of bFP (25ºC, $CDCl_3$)

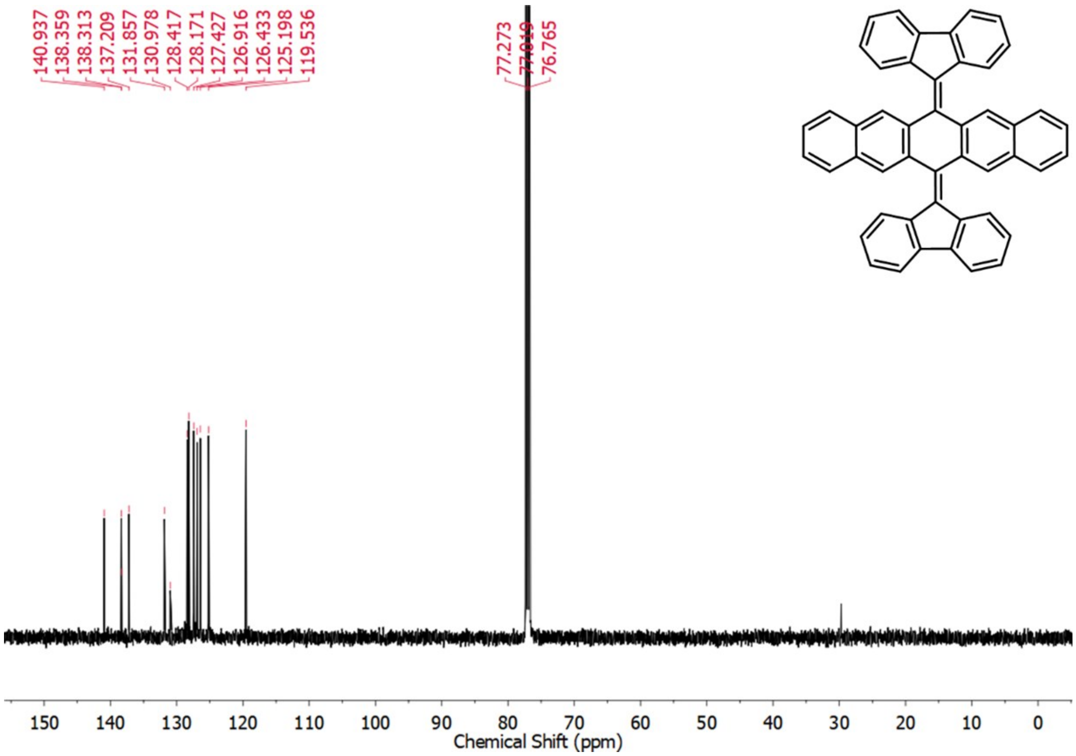

Hi-Resolution Mass Spectrum of bFP

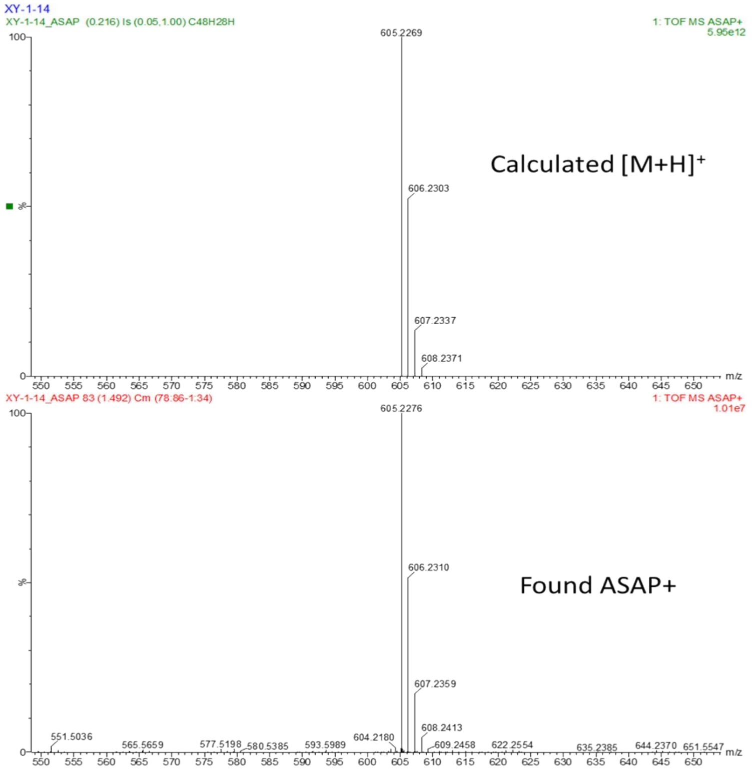

^{1}H NMR of 4 (25ºC, $CDCl_3$)

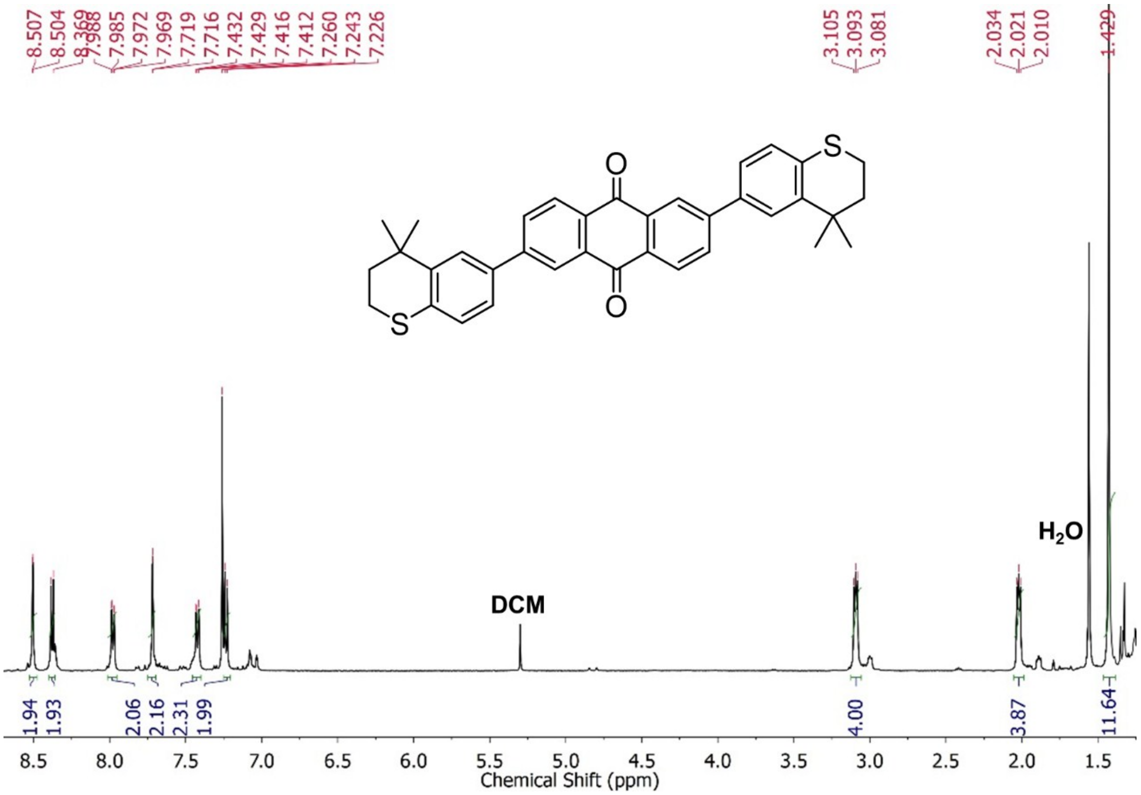

^{13}C NMR of 4 (25ºC, $CDCl_3$)

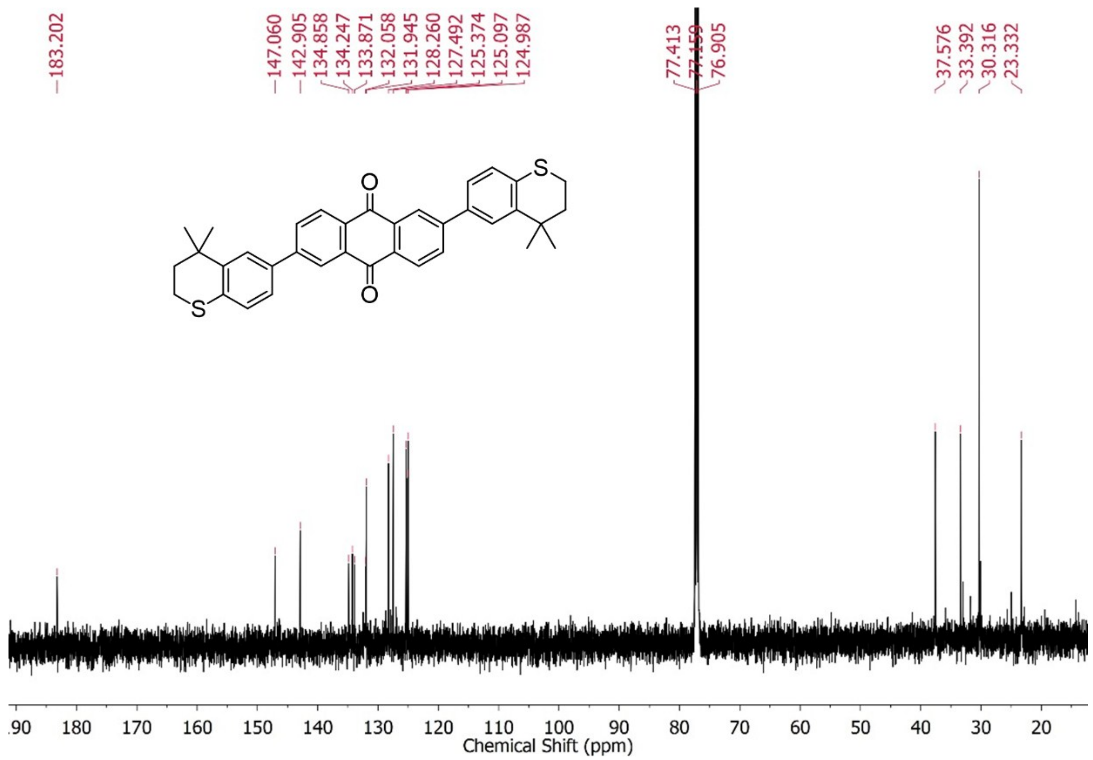

Hi-Resolution Mass Spectrum of 4

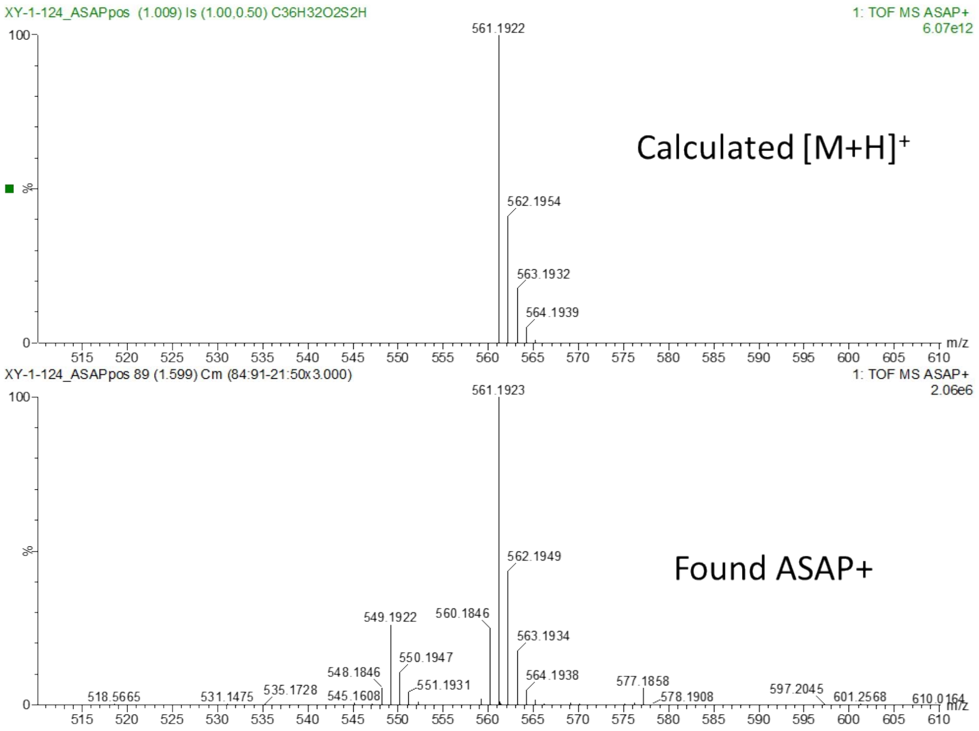

^{1}H NMR of 5 (25ºC, $CDCl_3$)

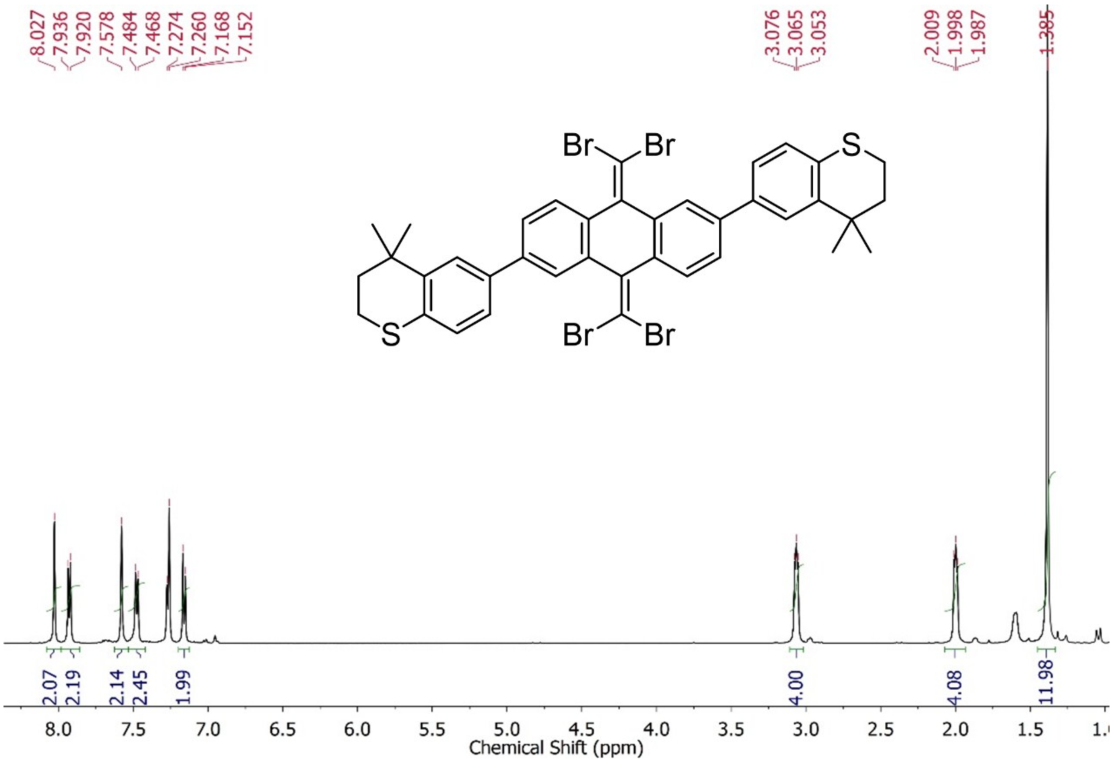

^{13}C NMR of 5 (25ºC, $CDCl_3$)

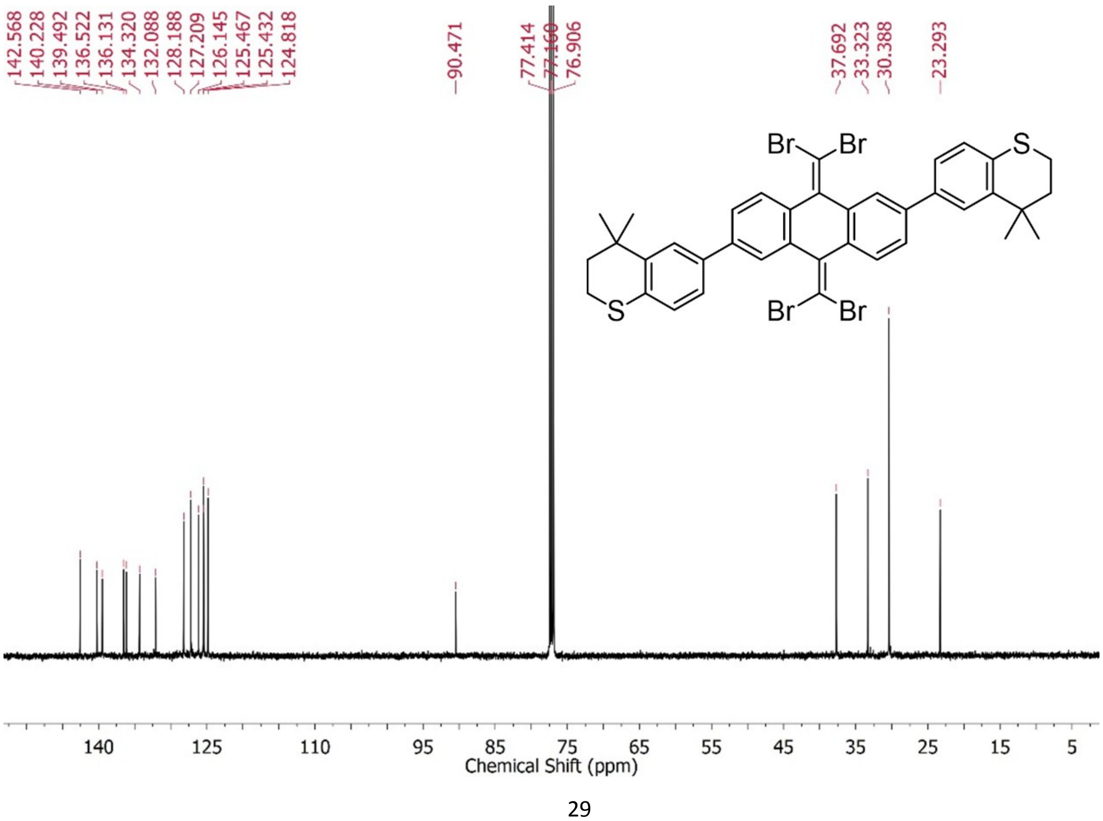

Hi-Resolution Mass Spectrum of 5

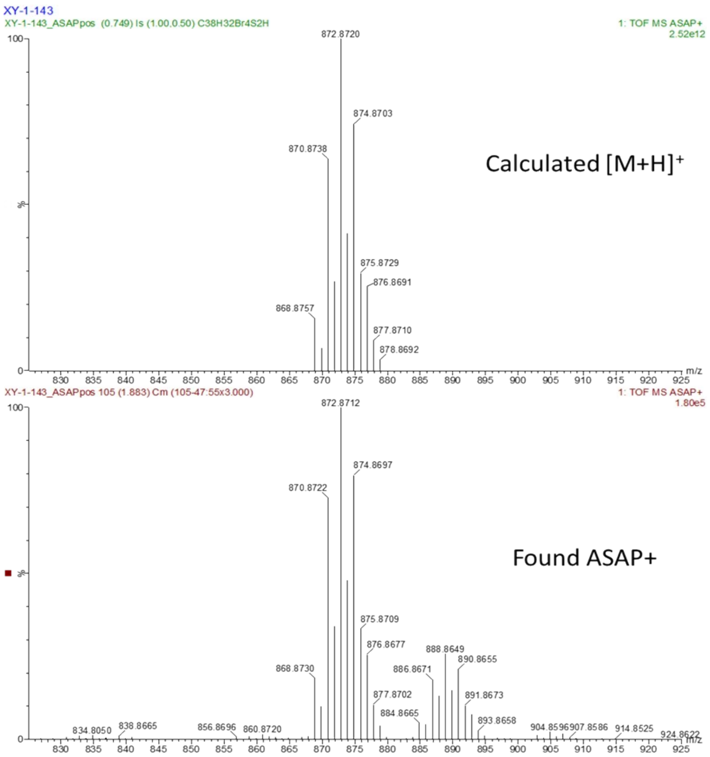

^{1}H NMR of bFA-t (25ºC, $CDCl_3$)

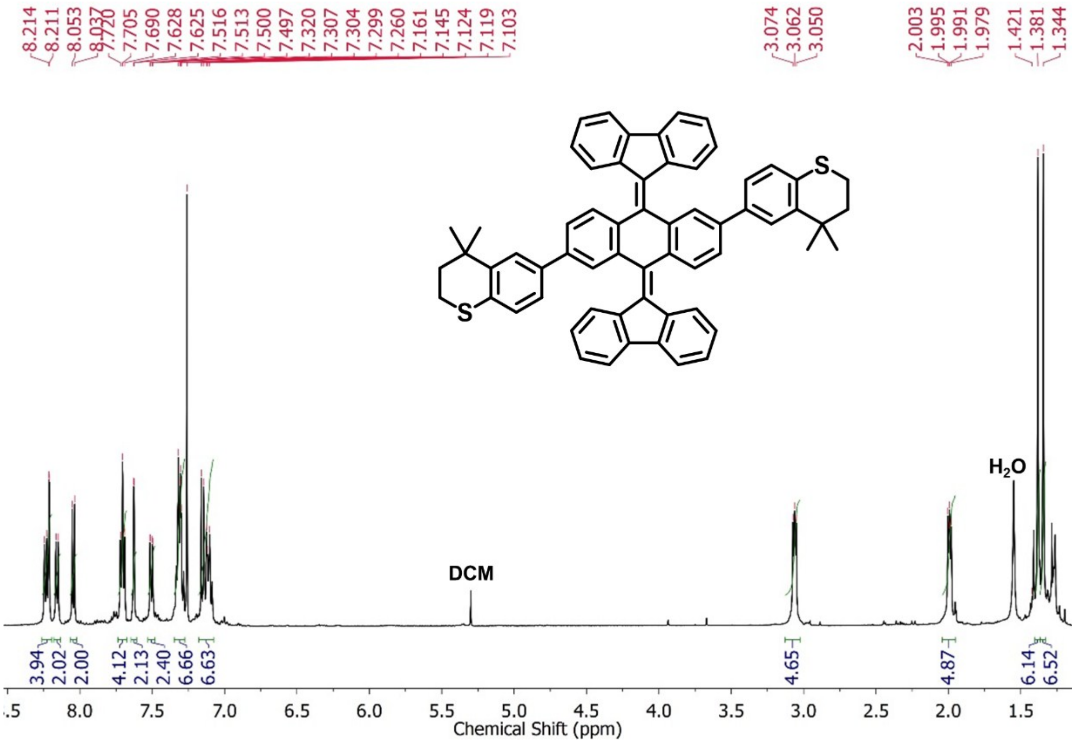

^{13}C NMR of bFA-t (25ºC, CD_2Cl_2)

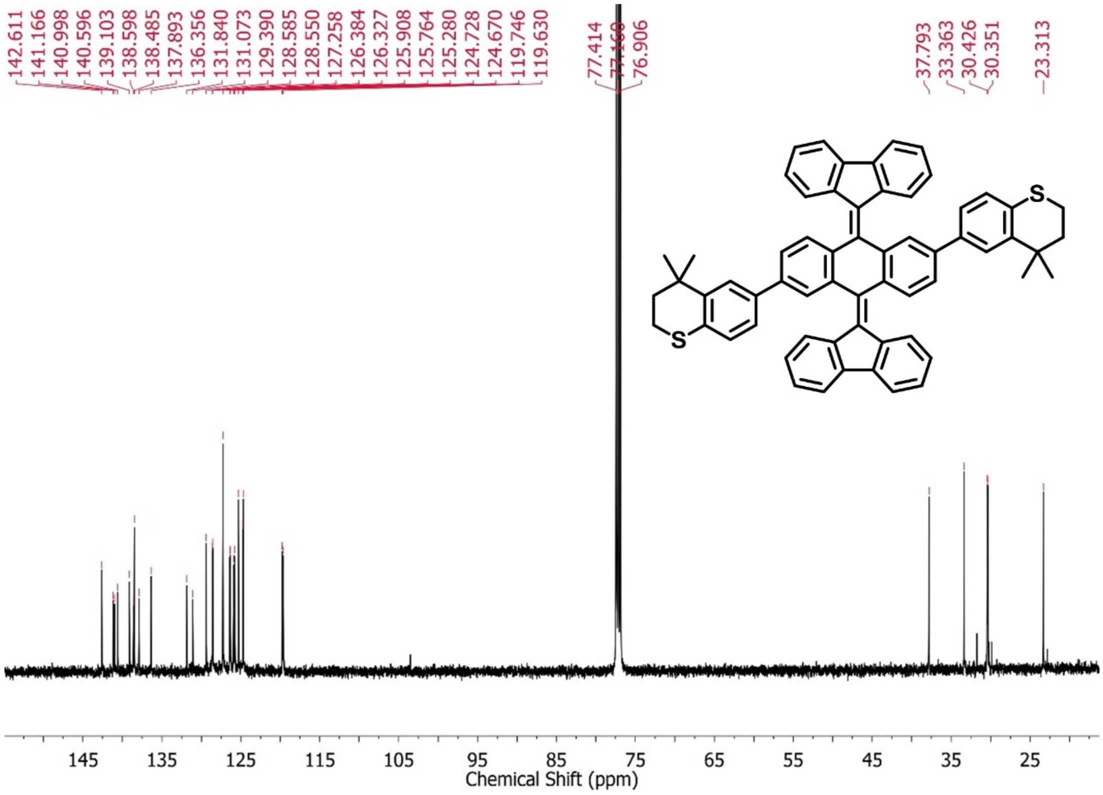

Hi-Resolution Mass Spectrum of bFA-t

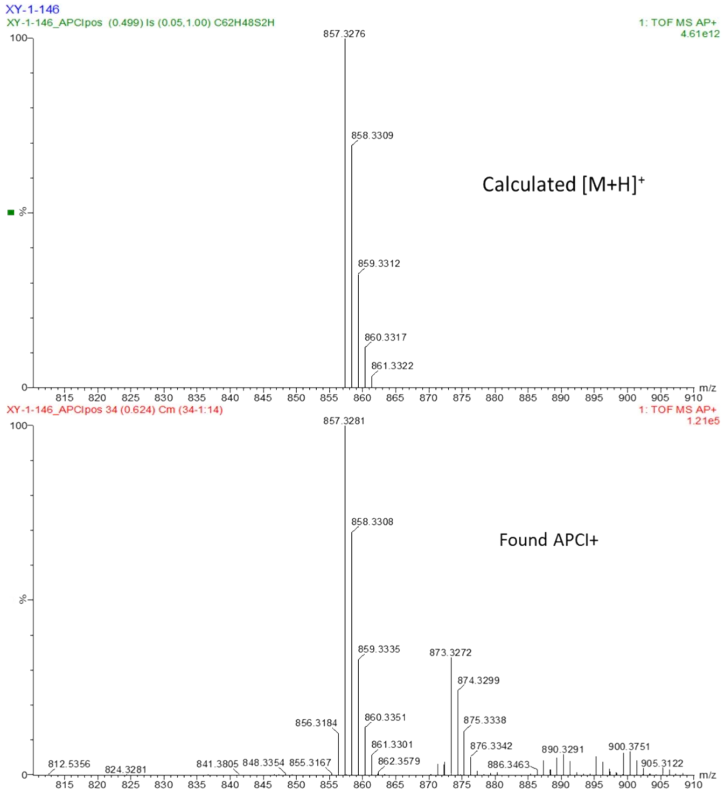

^{1}H NMR of bFAH-t (25°C, CD_2Cl_2)

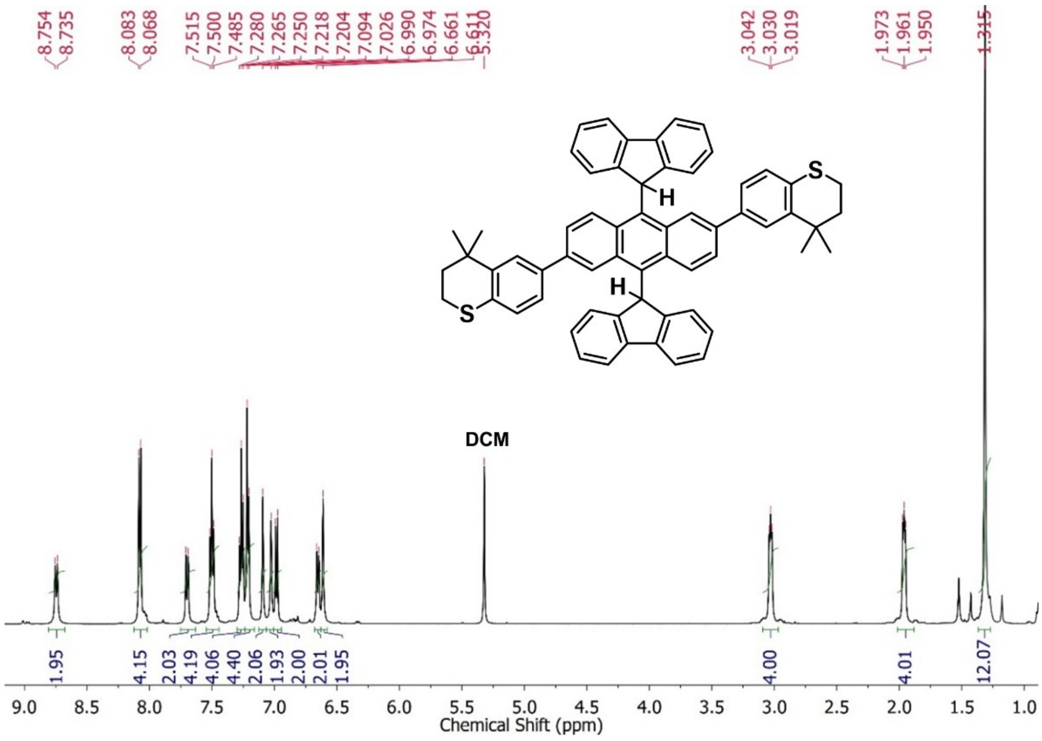

^{13}C NMR of bFAH-t (25°C, CD_2Cl_2)

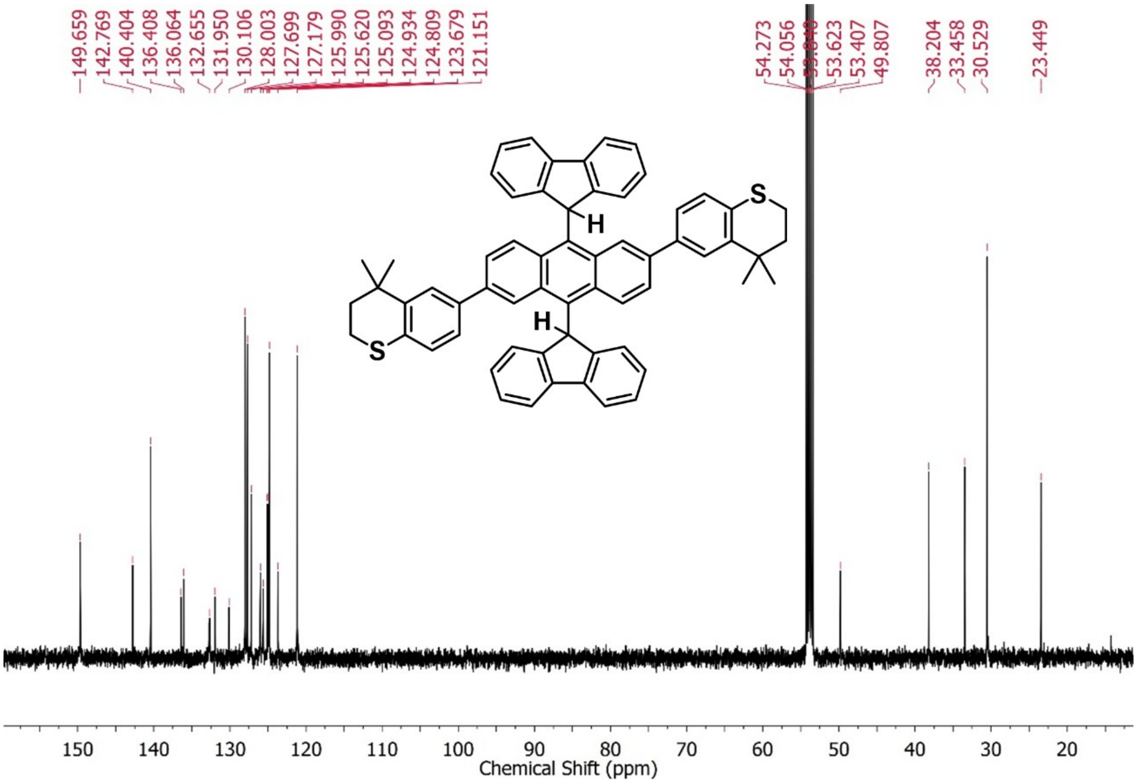

Hi-Resolution Mass Spectrum of bFAH-t

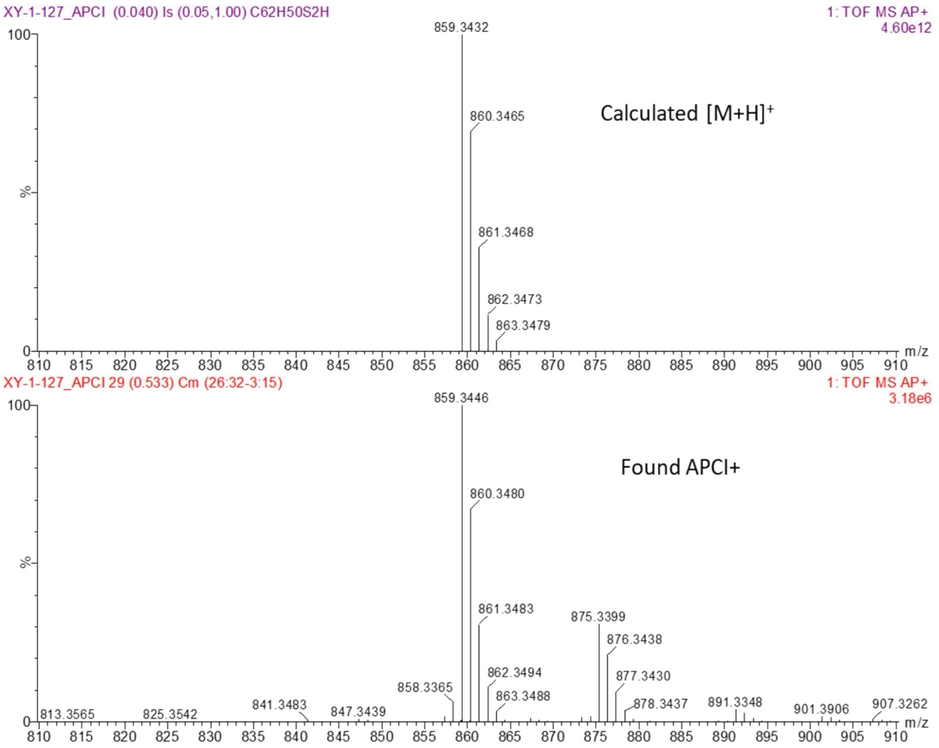

S7. References

1 *Gaussian 09, Revision D.01,* M. J. Frisch, G. W. Trucks, H. B. Schlegel, G. E. Scuseria, M. A. Robb, J. R. Cheeseman, G. Scalmani, V. Barone, G. A. Petersson, H. Nakatsuji, X. Li, M. Caricato, A. Marenich, J. Bloino, B. G. Janesko, R. Gomperts, B. Mennucci, H. P. Hratchian, J. V. Ortiz, A. F. Izmaylov, J. L. Sonnenberg, D. Williams-Young, F. Ding, F. Lipparini, F. Egidi, J. Goings, B. Peng, A. Petrone, T. Henderson, D. Ranasinghe, V. G. Zakrzewski, J. Gao, N. Rega, G. Zheng, W. Liang, M. Hada, M. Ehara, K. Toyota, R. Fukuda, J. Hasegawa, M. Ishida, T. Nakajima, Y. Honda, O. Kitao, H. Nakai, T. Vreven, K. Throssell, J. A. Montgomery, Jr., J. E. Peralta, F. Ogliaro, M. Bearpark, J. J. Heyd, E. Brothers, K. N. Kudin, V. N. Staroverov, T. Keith, R. Kobayashi, J. Normand, K. Raghavachari, A. Rendell, J. C. Burant, S. S. Iyengar, J. Tomasi, M. Cossi, J. M. Millam, M. Klene, C. Adamo, R. Cammi, J. W. Ochterski, R. L. Martin, K. Morokuma, O. Farkas, J. B. Foresman, and D. J. Fox,, Gaussian, Inc., Wallingford CT, 2016.

2 S. Pola, C. H. Kuo, W. T. Peng, M. M. Islam, I. Chao and Y. T. Tao, *Chem. Mater.*, 2012, **24**, 2566-2571.

3 Z. He, L. Shan, J. Mei, H. Wang, J. W. Y. Lam, H. H. Y. Sung, I. D. Williams, X. Gu, Q. Miao and B. Z. Tang, *Chem. Sci.*, 2015, **6**, 3538-3543.

4 C. Y. Chiu, B. Kim, A. A. Gorodetsky, W. Sattler, S. J. Wei, A. Sattler, M. Steigerwald and C. Nuckolls, *Chem. Sci.*, 2011, **2**, 1480-1486.
